# The magnitude and variability of neurocognitive performance in first-episode psychosis: a systematic review and meta-analysis of longitudinal studies

**DOI:** 10.1038/s41398-023-02718-6

**Published:** 2024-01-08

**Authors:** Ana Catalan, Robert A. McCutcheon, Claudia Aymerich, Borja Pedruzo, Joaquim Radua, Victoria Rodríguez, Gonzalo Salazar de Pablo, Malein Pacho, Jose Luis Pérez, Marco Solmi, Philip McGuire, Anthony J. Giuliano, William S. Stone, Robin M. Murray, Miguel Angel Gonzalez-Torres, Paolo Fusar-Poli

**Affiliations:** 1 Department of Neuroscience, University of the Basque Country UPV/EHU; Psychiatry Department. Basurto University Hospital; Biobizkaia Health Research Institute; Centro de Investigación en Red de Salud Mental. (CIBERSAM) Instituto de Salud Carlos III , OSI Bilbao-Basurto, Av. Montevideo 18, 48013 Bilbao, Spain; 2 Early Psychosis Interventions and Clinical-detection (EPIC) Lab, Department of Psychosis Studies, Institute of Psychiatry, Psychology & Neuroscience, King’s College London, London, UK; 3 Department of Psychosis Studies, Institute of Psychiatry, Psychology and Neuroscience, King’s College London, London, UK; 4 Department of Psychiatry. University of Oxford, Warneford Hospital, Headington, OX3 7JX UK; 5 Oxford Health NHS foundation trust, Oxford, UK; 6 Department of Neuroscience, University of the Basque CountryUPV/EHU. Psychiatry Department. Basurto University Hospital. BiBiobizkaia Health Research Institute. Centro de Investigaciónen Red de Salud Mental. (CIBERSAM), Instituto de Salud Carlos III, Avenida de Montevideo 18, 48013 Bilbao, Spain; 7 Psychiatry Department. Basurto University Hospital, OSI Bilbao-Basurto, Bizkaia, Spain; 8 Imaging of Mood- and Anxiety-Related Disorders (IMARD) Group, Institut d’Investigacions Biomèdiques August Pi i Sunyer (IDIBAPS), Mental Health Research Networking Center (CIBERSAM), Instituto de Salud Carlos III, University of Barcelona, Barcelona, Spain; 9 Department of Child and Adolescent Psychiatry, Institute of Psychiatry, Psychology & Neuroscience, King’s College London, London, UK; 10 Child and Adolescent Mental Health Services, South London and Maudsley NHS Foundation Trust, London, UK; 11 Department of Child and Adolescent Psychiatry, Institute of Psychiatry and Mental Health. Hospital General Universitario Gregorio Marañón School of Medicine, Universidad Complutense, IiSGM, CIBERSAM, Madrid, Spain; 12 Charité Universitätsmedizin Berlin, Department of Child and Adolescent Psychiatry, Berlin, Germany; 13 SCIENCES lab, Department of Psychiatry, University of Ottawa, Ottawa, ON Canada; 14 On Track: The Champlain First Episode Psychosis Program, Department of Mental Health, The Ottawa Hospital, Ottawa, ON Canada; 15 Ottawa Hospital Research Institute (OHRI) Clinical Epidemiology Program University of Ottawa, Ottawa, ON Canada; 16 School of Epidemiology and Public Health, Faculty of Medicine, University of Ottawa, Ottawa, ON Canada; 17 Department of Psychiatry, Beth Israel Deaconess Medical Center, Harvard Medical School, Boston, MA USA; 18 Department of Brain and Behavioral Sciences, University of Pavia, , Pavia, Italy; 19 Outreach and Support in South London (OASIS) service, South London and Maudsley NHS Foundation Trust, London, UK; 20 Department of Psychiatry and Psychotherapy, Ludwig-Maximilian-University Munich, Munich, Germany

**Keywords:** Prognostic markers, Diagnostic markers

## Abstract

Neurocognitive deficits are a core feature of psychotic disorders, but it is unclear whether they affect all individuals uniformly. The aim of this systematic review and meta-analysis was to synthesize the evidence on the magnitude, progression, and variability of neurocognitive functioning in individuals with first-episode psychosis (FEP). A multistep literature search was conducted in several databases up to November 1, 2022. Original studies reporting on neurocognitive functioning in FEP were included. The researchers extracted the data and clustered the neurocognitive tasks according to the seven Measurement and Treatment Research to Improve Cognition in Schizophrenia (MATRICS) domains and six additional domains. Random-effect model meta-analyses, assessment of publication biases and study quality, and meta-regressions were conducted. The primary effect size reported was Hedges g of (1) neurocognitive functioning in individuals at FEP measuring differences with healthy control (HC) individuals or (2) evolution of neurocognitive impairment across study follow-up intervals. Of 30,384 studies screened, 54 were included, comprising 3,925 FEP individuals and 1,285 HC individuals. Variability analyses indicated greater variability in FEP compared to HC at baseline and follow-up. We found better neurocognitive performance in the HC group at baseline and follow-up but no differences in longitudinal neurocognitive changes between groups. Across the 13 domains, individuals with FEP showed improvement from baseline in all studied domains, except for visual memory. Metaregressions showed some differences in several of the studied domains. The findings suggest that individuals with FEP have marked cognitive impairment, but there is greater variability in cognitive functioning in patients than in HC. This suggests that subgroups of individuals suffer severe disease-related cognitive impairments, whereas others may be much less affected. While these impairments seem stable in the medium term, certain indicators may suggest potential further decline in the long term for a specific subgroup of individuals, although more research is needed to clarify this. Overall, this study highlights the need for tailored neurocognitive interventions for individuals with FEP based on their specific deficits and progression.

## Introduction

Cognitive deficits are well-established, core features of psychotic disorders [1–3]. These impairments include neurocognitive deficits (i.e., attention, verbal learning and memory, working memory, and executive functions) and social cognitive deficits (related to processing and interpreting social information [1, 4–6]). The deficits are relatively stable during the illness, and nearly all cognitive deficits are comparable in magnitude across first episode psychosis (FEP) individuals and chronic schizophrenia [2], though recent studies show evidence for selective cognitive declines over time [7, 8]. Cognitive deficits are associated with difficulties in social functioning and a worse prognosis, [9–11] and are more predictive of social functioning than psychotic symptoms [12–14]

Whereas established non-pharmacological interventions for FEP patients usually include family interventions, psychoeducation, cognitive-behavioral therapy, and vocational interventions [15], cognitive impairments in early intervention services are generally undetected and undertreated. This is so despite the fact that attending to neurocognitive deficits through appropriate treatments has proved effective in improving social functioning [16].

However, we do not know whether the magnitude of these neurocognitive deficits changes over time and whether any potential deterioration is greater for one cognitive domain versus others. Two previous meta-analyses have studied the trajectory of neurocognition at baseline and follow-up [17, 18] and documented a lack of evidence for decline or improvement in general neurocognition. These did not, however, explicitly compare the trajectory in controls to that in patients. This is important due to the potential presence of practice effects – a stable trajectory in patients might, in fact, be significantly different from a trajectory of improvement in controls. In addition, these meta-analyses have not specifically looked at the variability of neurocognitive performance in patients.

In a recent work, we established that neurocognitive deficits are present before the onset of a psychotic disorder in several domains and implemented a method to harmonize the measurement of this deterioration across different tests quantifying the same neurocognitive domain [19]. The primary aim of this work was first to meta-analytically examine inter-patient variability to determine whether cognitive impairments are a relatively constant phenomenon across patients (i.e., a shift of the bell curve), or whether some individuals are severely affected, whereas others experience no disease-related impairment. Second, we aimed to examine any longitudinal change in neurocognitive functioning after the onset of psychosis in FEP individuals while considering the potential confounding effect of sociodemographic, clinical and methodological factors compared to healthy control (HC) subjects.

## Material and methods

The study protocol was registered on https://osf.io/r94t5/ and was conducted following the Preferred Reporting Items for Systematic Reviews and Meta-Analyses [20] (Supplementary Table 1), Meta-analysis of Observational Studies in Epidemiology (MOOSE) reporting guideline [21] (Supplementary Table 2), and Enhancing the Quality and Transparency of Health Research (EQUATOR) reporting guidelines [22]. We used studies that focused on individuals with first-episode psychosis, including those with a diagnosis of schizophrenia spectrum disorder (F20 to F29 according to ICD-11).

### Search strategy and selection criteria

A systematic search strategy was used to identify relevant articles, and a two-step literature search was implemented by two independent researchers (AC, BP) (search terms appended in Supplementary Methods 1). Web of Science database (Clarivate Analytics) was searched, incorporating the Web of Science Core Collection, BIOSIS Citation Index, KCI-Korean Journal Database, MEDLINE, Russian Science Citation Index, and SciELO Citation Index as well as Cochrane Central Register of Reviews, and Ovid/PsychINFO databases from inception to 1^st^ November 2022. Abstracts of identified articles were screened, and after excluding those not relevant, the full texts were assessed for eligibility. The references of previously published meta-analyses and systematic reviews and of the included articles were manually searched.

Studies were included if they (1) were original articles published in a peer-reviewed journal; (2) included individuals at FEP, defined according to established clinical criteria or validated psychometric scales (RDC, DSM, ICD, or equivalent with less than five years of illness evolution); (3) focused on neurocognitive tasks (Supplementary Table 3); (4) presented longitudinal data, with baseline and follow-up data; and (5) were published in English. Studies were excluded if they (1) were reviews, clinical cases, abstracts, conference proceedings, or study protocols; (2) used non-established criteria for defining FEP; (3) did not report meta-analyzable data; (4) reported only composite neurocognitive data (to avoid potentially spurious or pseudospecific results) [23]; (5) presented data of a neurocognitive intervention aimed to improve neurocognitive performance, or (6) overlapped on the same sample and neurocognitive task. When there were 2 or more overlapping studies, the one with the largest sample size was selected for analyses. In case of sample size overlapping, the most recent study was included.

### Outcome measures and data extraction

Four researchers (CA, BP, JLP, VR) independently extracted data from all identified studies (Supplementary Table 4). The databases were then cross-checked and discrepancies were resolved through consensus under the supervision of a senior researcher (AC). Consistent with our earlier meta-analysis [19], nine neurocognitive tasks were clustered into seven Measurement and Treatment Research to Improve Cognition in Schizophrenia (MATRICS) domains [24, 25], namely (1) processing speed, (2) attention or vigilance, (3) working memory, (4) verbal learning, (5) visual learning, (6) reasoning and problem-solving, and (7) social cognition (Supplementary Table 3). To ensure the comprehensiveness of our review, we also considered additional tasks that had been included in studies of this clinical population but not included in the more limited MATRICS framework (Supplementary Table 3). These tasks were categorized by senior experts (AG, WS, MP) into the following 8 domains: (1) general intelligence, (2) visuospatial ability, (3) verbal memory, (4) visual memory, (5) executive functioning, and (6) motor functioning. When the same study presented several follow-up time points, the last one was included in the analyses.

### Statistical analyses

The primary meta-analytical effect size measure was Hedges´ g, with positive values reflecting better functioning in HC individuals compared with FEP individuals, a greater impairment at baseline than in the follow-up in the FEP group, or a greater decline in FEP compared to HC.

For the main meta-analysis, each specific neurocognitive task was analyzed separately when at least 3 independent studies were available. We conducted 2 primary comparisons of neurocognitive functioning: (1) a cross-sectional meta-analysis of individuals at FEP vs HC individuals at baseline and follow-up, and (2) a meta-analysis of the difference in longitudinal change between individuals at FEP and HC. Additionally, we (3) performed a longitudinal meta-analysis to explore the evolution of neurocognitive impairment between baseline and follow-up solely in FEP. In each of these analyses we estimated both individual task effect sizes and the pooled effect sizes for individuals at FEP vs HC individuals across each of the 13 neurocognitive domains (Supplementary Methods 2) when more than one task was available. We did not include corrections for multiple comparisons, in accordance with the Cochrane´s recommendations [26].

For the analysis of change over time, the variance of the change score needs to be calculated if it is not reported. For studies not reporting the variance of the pre-post change, we calculated the mean change by subtracting the first from the second measure. We then calculated the variance via the standard formula:

$${{SD}}_{{change}}^{2}={{SD}}_{{baseline}}^{2}+{{SD}}_{{follow}-{up}}^{2}-2\,\cdot\, \rho \,\cdot\, {{SD}}_{{baseline}}\,\cdot\, {{SD}}_{{follow}-{up}}$$

This requires an estimation of the correlation coefficient, rho, between baseline and follow-up neurocognitive measures. Rho can be calculated from those studies reporting the mean (and SD) pre, post and change values; [27] these studies suggested rho to equal 0.65 (sensitivity analyses were performed at the limits of the 95% CI: 0.58 and 0.70 [28]).

In order to investigate the variability of cognition, we used previously established methods [29, 30]. Previous approaches assumed the nature of the relationship between their mean and SD [31], which could lead to biased estimates. To address these issues, we used a random-slope mixed-effects model (RSMM) to estimate differences in variability between groups (FEP and HC). Following Nakagawa et al. [30] and Maslej et al. [29], we used an unbiased estimator of the natural logarithm of the population SD and its sampling variance (Supplementary Methods 3).

For all meta-analyses, we used a random-effects model [32], as heterogeneity was expected to be high. Heterogeneity was assessed using the Q statistic and I^2^ index [33]. Publication biases were evaluated by visually inspecting funnel plots. When publication biases were detected, the trim-and-fill [34] method was used. Study quality was assessed using a modified the Newcastle-Ottawa Scale (NOS) version, previously validated in Clinical High Risk for Psychosis meta-analyses [19] (Supplementary Table 4). When at least 7 studies were available, meta-regressions evaluated the effect of several factors in merged domains.

All analyses were conducted within R 1.4.1106 [35], using the metafor package [36]. All tests were 2-sided, and significance was set at *P* < 0.05.

## Results

### Characteristics of the database

**Fig. 1:**
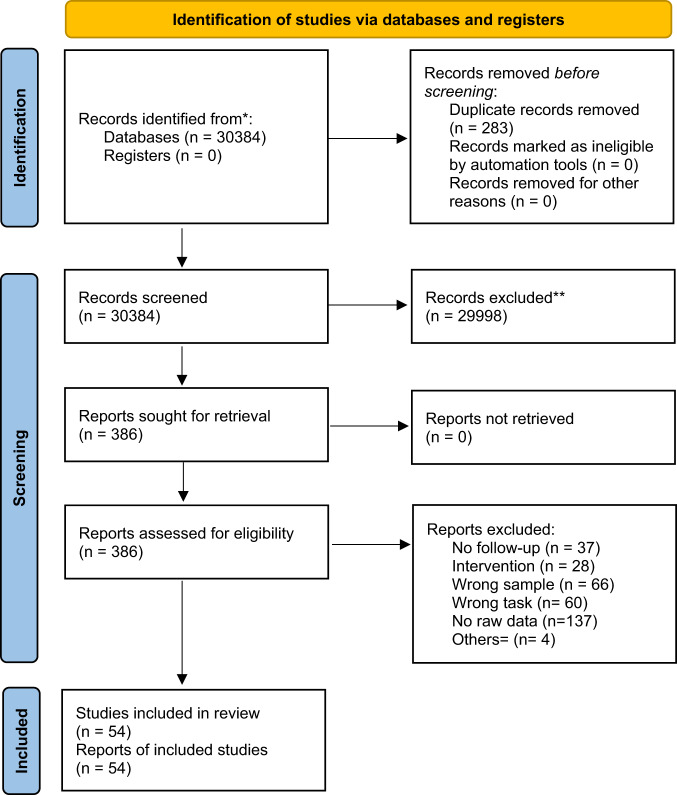
Flow-chart of meta-analysis.

Of 30384 studies screened, 386 were retrieved for full-text assessment and 54 were included (Fig. 1; Supplementary Table 5), comprising 3925 FEP individuals (mean = 26.00 years, SD = 4.44, 68.60% male) and 1285 HC individuals (mean = 25.25, SD = 5.73, 57.19% male). The mean (SD) education was 12.35 (SD = 2.02) years for FEP individuals and 13.63 (SD = 2.56) for HC individuals.

At baseline, 78.22% of FEP individuals were known to be treated with antipsychotic (AP) medication (at any dosage); 18 studies did not offer data about AP treatment. The mean follow-up time was 33.82 (SD = 42.96) months (from 2 to 240 months), while the median was 2 years.

### Variability of neurocognitive functioning: FEP vs HC

**Fig. 2:**
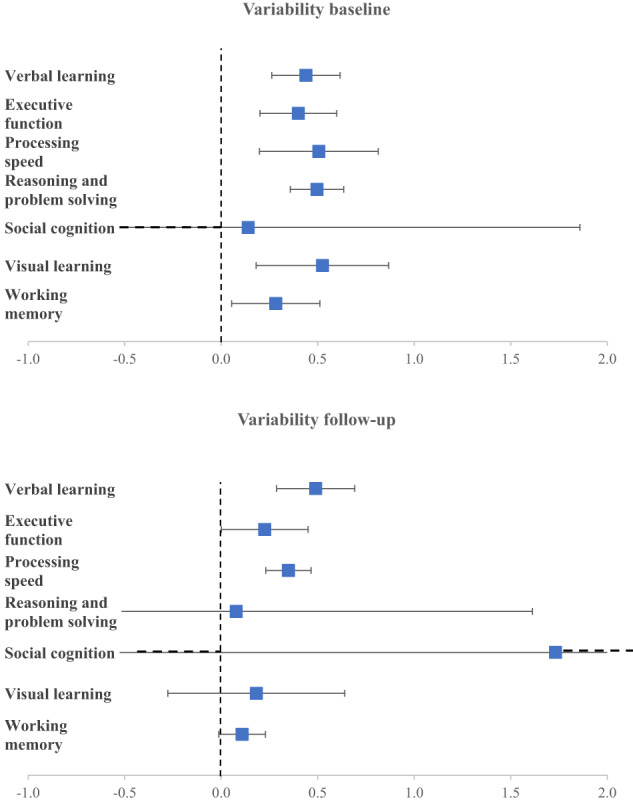
Variability between HC and FEP in the different neurocognitive domains at baseline and follow-up. Values greater than 0 indicate greater variability in FEP group.

At baseline assessment, the FEP group presented greater variability than HC across several neurocognitive domains: visual learning (ES = 0.52, SE = 0.14, *p* < 0.01), processing speed (ES = 0.50, SE = 0.14, *p* < 0.01), reasoning and problem-solving (ES = 0.50, SE = 0.03, *p* < 0.01), verbal learning (ES = 0.44, SE = 0.08), executive functioning (ES = 0.40, SE = 0.09, *p* < 0.0001), and working memory (ES = 0.28, SE = 0.10) (Fig. 2). At follow-up, the FEP group presented greater variability than HC across these neurocognitive domains: verbal learning (ES = 0.49, SE = 0.09, *p* < 0.001), processing speed (ES = 0.35, SE = 0.05, *p* < 0.0001), and executive functioning (ES = 0.22, SE = 0.10, *p* = 0.049) (Fig. 2). The variability for each individual task is detailed in the supplementary material (Supplementary Table 6).

### Neurocognitive functioning in FEP individuals compared with HC individuals

#### At baseline

Within the 13 domains (Supplementary Fig. 1, Supplementary Table 7), FEP individuals performed worse than HC individuals in the following tasks (in descending order of magnitude): California Verbal Learning Test-I immediate recall (CVLT-I immediate recall) (*g* = 2.05; 95% CI, 0.03–4.08), Brief Visuospatial Memory Test—Revised (BVMT-R) (*g* = 1.90; 95% CI, 0.24–3.56), Neuropsychological Assessment Battery: Mazes (NAB Mazes) (*g* = 1.70; 95% CI, 0.18–3.22), Wechsler Adult Intelligence Scale IV Digit Symbol (WAIS-IV Digit Symbol) (*g* = 1.52; 95% CI, 1.30–1.74), Rey Auditory Verbal Learning Test 1-5 (RAVLT) (*g* = 1.47; 95% CI, 0.76–2.18), RAVLT delayed recall (*g* = 1.38; 95% CI, 1.06–1.71), Trail Making Test A (TMT-A) (*g* = 1.24; 95% CI, 0.34–2.14), California Verbal Learning Test-I 1-5 (CVLT-I 1-5) (*g* = 1.05; 95% CI, 0.44–1.66), CVLT-I delayed recall (*g* = 1.01; 95% CI, 0.48–1.55), Animal Fluency (*g* = 1.01; 95% CI, 0.51–1.5), Letter Number Sequencing Test (LNST) (*g* = 1.01; 95% CI, 0.84–1.17), Hopkins Verbal Learning Test-Revised (HVLT-R) (*g* = 0.96; 95% CI, 0.59–1.32), Category fluency (*g* = 0.85; 95% CI, 0.29–1.41), The Mayer-Salovey-Caruso Emotional Intelligence Test (MSCEIT) (*g* = 0.83; 95% CI, 0.63–1.02), TMT-B (*g* = 0.78; 95% CI, 0.56–0.99), Rey-Osterrieth Complex Figure (ROCF) delayed recall (*g* = 0.74; 95% CI, 0.38–1.10), WAIS-IV Digit Span Backwards (*g* = 0.70; 95% CI, 0.49–0.92), Controlled Oral Word Association Test (COWAT) (*g* = 0.69; 95% CI, 0.33–1.05), Wisconsin Card Sorting Test (WCST) perseverative errors (*g* = 0.62; 95% CI, 0.45–0.79), and WCST categories (*g* = 0.57; 95% CI, 0.43–0.70), WAIS-IV Digit Span Forwards (*g* = 0.40; 95% CI, 0.19–0.61). On the Grooved Pegboard Test-dominant hand (*g* = −0.67; 95% CI, −0.87 to −0.47), the FEP group performed better than HC.

There were no differences in the Stroop Color and Word Test (SCWT) Word, SCWT color, SCWT Color-Word, Logical Memory (LM) immediate recall, Wechsler Memory Scale Visual Memory (WMS VM) immediate recall, LM delayed recall, and WCST perseverative responses (Supplementary Fig. 1).

**Fig. 3:**
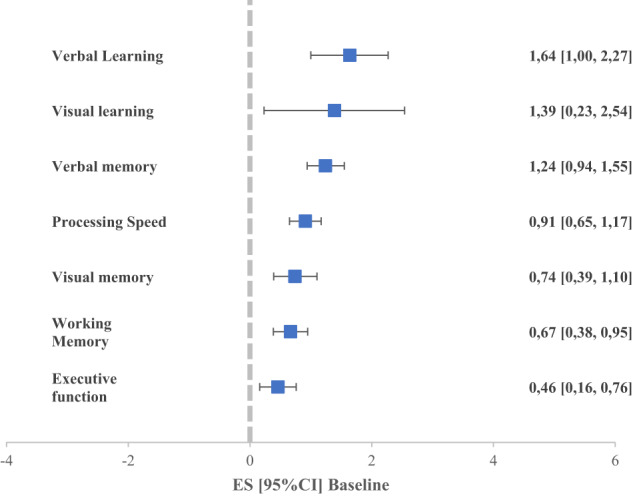
Comparison of neurocognitive functioning between FEP and HC at baseline (domains merged). Values greater than 0 indicate greater deficits in FEP group.

When all neurocognitive tasks were pooled across the 7 broader neurocognitive domains (Fig. 3, Supplementary Table 8), FEP individuals performed more poorly than HC individuals across all domains (in decreasing order of magnitude): verbal learning (*g* = 1.64; 95% CI, 1.00–2.27), visual learning (*g* = 1.39; 95% CI, 0.23–2.54), verbal memory (*g* = 1.24; 95% CI, 0.15–0.94), processing speed (*g* = 0.91; 95% CI, 0.13–0.65), visual memory (*g* = 0.74; 95% CI, 0.39–1.10), working memory (*g* = 0.67; 95% CI, 0.38–0.95), and executive function (*g* = 0.46; 95% CI, 0.15–0.16).

#### At follow-up

Within the 13 domains (Supplementary Fig. 2, Supplementary Table 8), FEP individuals performed worse than HC individuals on the following tasks (in descending order of magnitude): WAIS-IV Digit Symbol (*g* = 1.79; 95% CI, 1.21–2.37), CVLT-II immediate recall (*g* = 1.71; 95% CI, 0.2–3.16), BVMT-R (*g* = 1.37, 95% CI, 0.37–2.36), SCWT Word (*g* = 1.21, 95% CI, 0.92–1.51), LNST (*g* = 1.19, 95% CI, 0.46–1.92), Category Fluency (*g* = 1.14, 95% CI, 0.90–1.38), CVLT 1-5 (*g* = 1.02, 95% CI, 0.70–1.34), CVLT delayed recall (*g* = 1.02, 95% CI, 0.70–1.35), HVLT-R (*g* = 0.84, 95% CI, 0.56–1.13), NAB Mazes (*g* = 0.81, 95% CI, 0.25–1.36), TMT-A (*g* = 0.79, 95% CI, 0.41–1.17), COWAT (*g* = 0.77, 95% CI, 0.58–0.96), TMT-B (*g* = 0.69, 95% CI, 0.45–0.94), WAIS-IV Digit Span Backwards (*g* = 0.67, 95% CI, 0.30–1.04), WCST perseverative errors (*g* = 0.55, 95% CI, 0.18–0.91), and WCST categories (*g* = 0.50, 95% CI, 0.30–0.69).

There were no differences in the SCWT Color, SCWT Color-Word, Logical Memory immediate recall, WMS VM immediate recall, MSCEIT, WMS LM delayed recall, WMS VR delayed recall and WCST perseverative responses (Supplementary Figure 2).

**Fig. 4:**
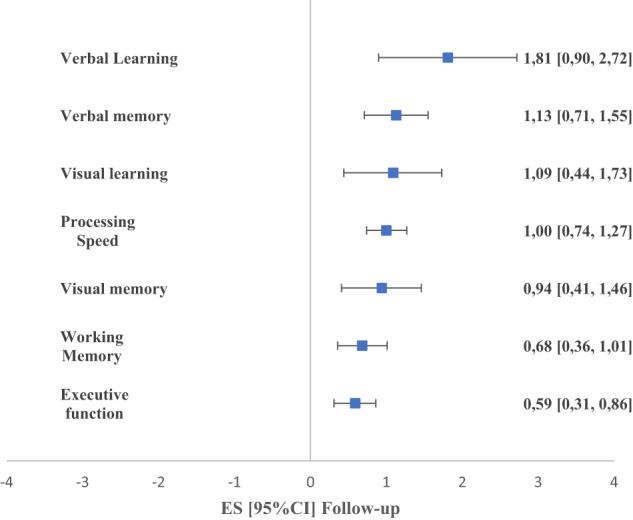
Comparison of neurocognitive functioning between FEP and HC at follow-up (domains merged). Values greater than 0 indicate greater deficits in FEP group.

When all neurocognitive tasks were pooled across the 7 broader neurocognitive domains (Fig. 4, Supplementary Table 9), FEP individuals performed more poorly than HC individuals across all domains (in decreasing order of magnitude): verbal learning (*g* = 1.81; 95% CI, 0.90–2.72), verbal memory (*g* = 1.13; 95% CI, 0.71–1.55), visual learning (*g* = 1.09; 95% CI, 0.44–1.73), processing speed (*g* = 1.00; 95% CI, 0.74–1.27), visual memory (*g* = 0.94; 95% CI, 0.41–1.46), working memory (*g* = 0.68; 95% CI, 0.36–1.01), and executive function (*g* = 0.59; 95% CI, 0.31–0.86).

#### Differences in the evolution of cognitive performances

**Fig. 5:**
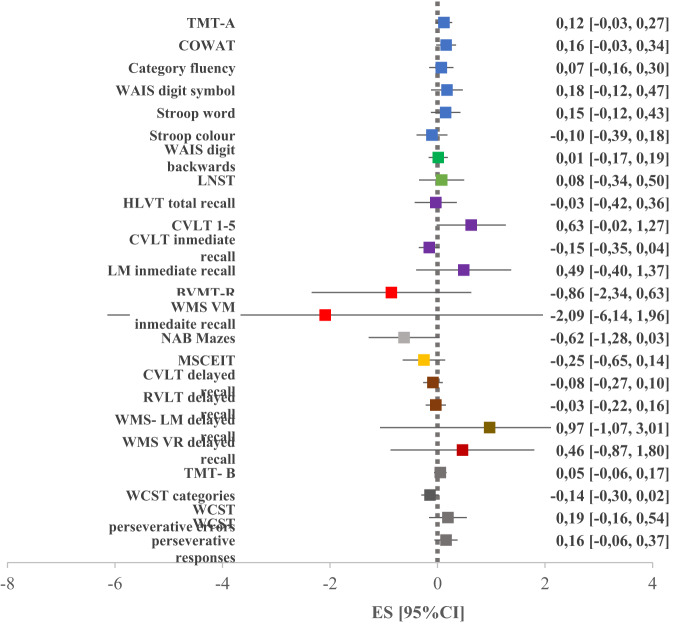
Differences in baseline to follow-up changes of neurocognitive functioning between FEP and HC. Values greater than 0 indicate greater deficits in FEP group. TMT Trail Making Test, COWAT Controlled Oral Word Association Test, WAIS Weschler Adult Intelligence Scale, LNST Letter Number Sequencing Test, HVLT Hopkins Verbal Learning Test, CVLT California Verbal Learning Test, LM logical memory, BVMT-R Brief Visuospatial Memory Test-Revised, WMS VM Weschler Memory Scale Visual Memory, NAB Neuropsychological Assessment Battery, MSCEIT Mayer-Salovey-Caruso Emotional Intelligence Test, RAVLT-R Rey Auditory Verbal Learning Test-Revised, WMS VR Weschler Memory Scale Visual Reproduction, WCST Wisconsin Card Sorting Test.

Baseline to follow-up changes in neurocognitive performance did not differ significantly between FEP and HC in any of the explored tasks (Fig. 5). The neurocognitive profiles of the FEP group exhibited greater variability at both baseline and follow-up when compared to the HC group. However, when observing the change in neurocognitive performance from baseline to follow-up, both FEP and HC patients showed consistent patterns with no differences between the groups. However, when observing the change in neurocognitive performance from baseline to follow-up, both FEP and HC patients showed consistent patterns with no differences between the groups.

#### Metaregressions

At baseline, an older age and longer follow-up were related to greater impairment in FEP group in verbal learning (ß = 0.28; 95% CI, 0.12–0.44; ß = 0.08; 95% CI, 0.05–0.11), verbal memory (ß = 0.74; 95% CI, 0.49–0.98; ß = 0.17; 95% CI, 0.12–0.21), and visual memory (ß = 0.83; 95% CI, 0.51–1.15; ß = 0.06; 95% CI, 0.03–0.10). Likewise, a lower quality score was related to decreased performance in verbal learning (ß = −1.96; 95% CI, −2.7 to −1.22), visual learning (ß = −2.57; 95% CI, −4.04 to −1.11), visual memory (ß = −3.41; 95% CI, −4.57 to −2.25), and verbal memory (ß = −4.35; 95% CI, −5.68 to −3.02). And a longer follow-up was also related to worse functioning in visual learning (ß = 0.09; 95% CI, 0.04–0.14) (Supplementary Table 10) at baseline.

An older age and longer follow-up were related to greater impairment in the FEP group in verbal learning both at baseline and at follow-up (ß = 0.30; 95% CI, 0.14–0.45; ß = 0.08; 95% CI, 0.06–0.10), and with a greater impairment in verbal memory at follow-up (ß = 0.66; 95% CI, 0.40–0.92; ß = 0.16; 95% CI, 0.11–0.21). And older age was also associated with decreased functioning in visual memory (ß = 1.20; 95% CI, 0.59–1.81) at follow-up. Likewise, lower quality scores were related to decreased performance in verbal memory (ß = −3.90; 95% CI, −5.40 to −2.47), visual memory (ß = −4.84; 95% CI, −7.07 to −2.62), and executive function (ß = −0.31; 95% CI, 0.02–0.60) at follow-up (Supplementary Table 10).

### Neurocognition performance in FEP group baseline vs follow-up

Within the 13 domains (Supplementary Table 11), FEP individuals performed worse at the baseline assessment than at the follow-up on the following tasks (in descending order of magnitude):

WMS paired associates (*g* = 0.47; 95% CI, 0.23–0.72), IQ Performance (*g* = 0.44; 95% CI, 0.25–0.63), TMT-B (*g* = 0.42; 95% CI, 0.02–0.82), WCST perseverative errors (*g* = 0.36; 95% CI, 0.16–0.56), WMS-III: Spatial Span (*g* = 0.36; 95% CI, 0.15–0.57), RAVLT delayed recall (*g* = 0.34; 95% CI, 0.23–0.44), CVLT-II immediate recall (*g* = 0.31; 95% CI, 0.20–0.43), RAVLT 1-5 trials (*g* = 0.31; 95% CI, 0.036–0.58), CVLT-II 1-5 (*g* = 0.31; 95% CI, 0.17–0.45), HVLT-R (*g* = 0.30; 95% CI, 0.14–0.46), CVLT-II delayed recall (*g* = 0.30; 95% CI, 0.20–0.41), IQ (*g* = 0.27; 95% CI, 0.08–0.45), CPT-reaction time (*g* = 0.27; 95% CI, 0.14–0.39), LM immediate recall (*g* = 0.26; 95% CI, 0.15–0.38), WCST-IV perseverative responses (*g* = 0.25; 95% CI, 0.10–0.41), verbal IQ (*g* = 0.24; 95% CI, 0.01–0.47), TMT-A (*g* = 0.24; 95% CI, 0.10–0.38), WCST categories (*g* = 0.23; 95% CI, 0.16–0.30), CPT- identical pairs (IP) d’ (*g* = 0.20; 95% CI, 0.01–0.38), Animal Fluency (*g* = 0.19; 95% CI, 0.01–0.37), COWAT (*g* = 0.17; 95% CI, 0.07–0.27), WAIS-IV Digit Backwards (*g* = 0.15; 95% CI, 0.05–0.25), and Letter Fluency (*g* = 0.14; 95% CI, 0.01–0.27). There were no differences in the rest of the studied tasks.

When all neurocognitive tasks were pooled across the 7 broader neurocognitive domains (Supplementary Fig. 3), FEP individuals performed more poorly at baseline than at follow-up across all domains except for visual memory (in decreasing order of magnitude): general intelligence (*g* = 0.91; 95% CI, 0.38–1.45), processing speed (*g* = 0.86; 95% CI, 0.67–1.05), working memory (*g* = 0.79; 95% CI, 0.44–1.139, verbal learning (*g* = 0.64; 95% CI, 0.42–0.86), motor function (*g* = 0.60; 95% CI, 0.06 to 1.14), visual learning (*g* = 0.56; 95% CI, 0.04–1.07), executive function (*g* = 0.46; 95% CI, 0.14–0.79), verbal memory (g = 0.35; 95% CI, 0.13–0.57), attention/vigilance (*g* = 0.20; 95% CI, 0.01–0.38).

#### Metaregressions

Male sex was associated with a greater improvement at follow-up in processing speed (ß = 0.02; 95% CI, 0.002–0.04; ß = 0.17) in the FEP group; and with lower improvement in verbal learning (ß = −0.03; 95% CI, −0.05 to −0.002) and verbal memory (ß = −0.02; 95% CI, −0.04 to −0.002). Older age was related to a greater improvement in visual learning in the FEP group (ß = 0.18; 95% CI, 0.03–0.34). Positive psychotic symptoms at baseline were related to greater improvement in working memory (ß = 0.07; 95% CI, 0.04–0.14). A longer follow-up correlated with lower improvement in verbal memory (ß = −0.01; 95% CI, −0.02 to −0.001). Furthermore, finally, the quality of included studies influenced the improvement in verbal memory (ß = 0.28; 95% CI, 0.08–0.49) and visual learning (ß = −0.54; 95% CI, −1.02 to −0.06) (Supplementary Table 12).

### Heterogeneity, study quality and publication bias

Heterogeneity across the studies varied from small to high (Supplementary Tables 7, 8 and 11). In terms of study risk of bias, NOS scores ranged from 4 to 8 (mean = 6.42; median = 6). Publication biases are reported in Supplementary Tables 7, 8, 11 and Supplementary Fig. 4.

## Discussion

The present study aimed to analyze whether variability neurocognitive functions differ between FEP and HC groups and if there are significant neurocognitive differences between the FEP group and HC individuals over time. Greater variability in FEP compared to HC was shown in some of the neurocognitive domains at baseline and follow-up (verbal learning, executive function and processing speed), while reasoning and problem-solving and visual learning showed higher variability in the FEP group compared to HC only at the baseline. Interestingly, those domains with greater variability in the FEP population compared with HC were also among the ones that showed greater decline among the FEP samples. Other authors [37, 38] have described evidence for neurocognitive variability in FEP, but to our knowledge, this is the first time that it has been distinguished from the general variability seen in healthy populations. Variability in the FEP group may be indicative of a subtype of patients with psychosis likely to demonstrate more decline in neurocognitive domains and thus might benefit from earlier and more intensive treatments from their period of disorder onset.

Contrary to the prevailing neurodegenerative hypothesis of psychosis, our research found no evidence of cognitive function decline in individuals in the FEP group. Moreover, these subjects showed an improved neurocognitive performance between baseline and follow-up in certain tasks. These results are consistent with previous studies [18, 39] questioning the neurodegenerative hypothesis of psychosis, which remains a highly debatable topic, partly due to the short follow-up duration in most studies, which makes difficult to reach definitive conclusions.

Nevertheless, our meta-regressions reveal a correlation between longer follow-up periods and greater cognitive decline, particularly in verbal and visual learning and memory domains. This finding, along with the evidence linking longer follow-up periods to more significant cognitive deficits, supports the notion of worse outcomes for certain psychotic patients in longer-term follow-ups. [40] While earlier meta-analyses [17, 18] established no increasing deterioration over time, they were limited by fewer studies and did not analyze group differences on individual tasks nor the variability of patient-control group differences. In contrast, more recent meta-analyses [18] described only modest improvements over time in the FEP group, with an effect size identical to that in HC, suggesting these changes might be an artefact of practice rather than genuine recovery. Our own meta-analysis corroborates these small improvements across specific domains and tasks, including social cognition, although these improvements were not significantly different from those in HC despite this group already performing significantly better across all tasks.

One possible explanation is that the magnitude of practice effects is greater for studies with short follow-up periods [41–43] is high. Although we have included studies with 20 years of follow-up [40], the median follow-up time was two years. The most consistent improvements were observed in tasks with significant practice effects (i.e., WCST, memory tasks [43]), while tasks with lesser practice effects such as letter fluency [44] showed less consistency in improvements. Notably, no enhancements were seen in visual memory tasks, suggesting a more stable deficit in this area. The visual system has been related to the transition to psychosis [45], and although the exact mechanism is unknown, many studies indicate that visual pathways could be related to psychosis onset [46, 47]. Similarly, none of the motor functioning tasks showed improvement at follow-up, which aligns with the fact that the FEP group presented a better performance than the HC group on some of these tasks (e.g., Grooved Pegboard Test). Motor coordination has been consistently linked to neurodevelopment alterations in individuals with psychosis and even children of parents with psychosis [48], and has been proposed as a sensorimotor dimension that cuts across psychopathology and that has causal and prognostic value as a psychosis endophenotype [49]. Concurrently, the use of antipsychotic medication is related to motor alterations [50].

Symptomatic remission and recovery could also influence these results, as most studies show a clinically significant improvement in the psychotic symptoms from the onset of the illness. As for the use of antipsychotic medication, studies show conflicting results, with some evidence for improvements in neurocognitive performance in FEP [51], while other studies do not provide evidence of improvement [52]. In our study, the use of antipsychotics was no related to change in neurocognitive performance, and we only observed a positive correlation between psychotic symptoms at baseline and improvement in working memory at follow-up. However, not all the included studies provided data on psychopathological status, which limits the results. Several factors could explain this. FEP patients with higher positive symptoms are treated earlier patients with negative symptoms [53]. Furthermore, several studies have linked the improvement in some cognitive domains with the use of antipsychotics [51], especially working memory [54], but this improvement cannot be generalized to all domains.

As expected, we found differences between FEP and HC individuals at baseline and follow-up, with the HC group presenting a better neurocognitive performance, although these differences seem similar at the two assessment points. There were especially significant differences in verbal and visual domains.

Although our findings lend greater support for a neurodevelopmental rather than a neurodegenerative model of cognitive deficits in schizophrenia, given the relatively short follow-up periods of most included studies, this remains an open question, and longer-term studies do suggest some deterioration may occur over longer intervals, at least for subgroups of individuals [40]. However, the lifespan timing and developmental trajectories of cognitive abnormalities in schizophrenia spectrum disorders require ongoing and better characterization.

Limitations of this meta-analysis include differences in study methodologies, such as variable and limited follow-up intervals, with a median follow-up of two years. Furthermore, many studies did not report variables that might have affected neurocognition, such as positive/negative symptoms, role and social functioning, pharmacological treatment or cannabis and other substance use, making it difficult to establish to what extent the change in the neurocognitive profile is due to the disorder itself or other factors. The decision to prioritize the inclusion of a limited number of studies with longitudinal data, as opposed to a larger pool of available cross-sectional studies, may be seen as a trade-off when addressing certain aspects of our research, such as the examination of cross-sectional findings, including variability and comparisons between the FEP and HC groups at baseline. Finally, the search terms included in our work were quite wide, and this could limit its replicability. Advantages of this study include being the first meta-analysis of longitudinal cognitive change in FEP, examination of confounding factors, and homogenous distribution of effect sizes. Another important issue is the inclusion of some affective diagnosis participants in the same samples, and some studies did not distinguish between the performances of those individuals with schizophreniform disorders and others [55, 56]. Yet another issue is related to high diagnostic instability and significant symptom heterogeneity attributed to patients with FEP. However, one meta-analysis showed relative diagnostic stability in FEP subjects [57].

In summary, we find that cognitive deficits are pronounced in first-episode patients but vary moderately in their severity among individuals and show no evidence of progression during the initial years of the illness.

### Supplementary information

supplementary material

## References

[1] Bora E, Murray RM (2014). Meta-analysis of cognitive deficits in ultra-high risk to psychosis and first-episode psychosis: do the cognitive deficits progress over, or after, the onset of psychosis?. Schizophr Bull.

[2] Mesholam-Gately RI, Giuliano AJ, Goff KP, Faraone SV, Seidman LJ (2009). Neurocognition in first-episode schizophrenia: a meta-analytic review. Neuropsychology.

[3] McCutcheon RA, Keefe RSE, McGuire PK (2023). Cognitive impairment in schizophrenia: aetiology, pathophysiology, and treatment. Mol Psychiatry.

[4] Bortolato B, Miskowiak KW, Koehler CA, Vieta E, Carvalho AF (2015). Cognitive dysfunction in bipolar disorder and schizophrenia: a systematic review of meta-analyses. Neuropsychiatr Dis Treat.

[5] Dickinson D, Ramsey ME, Gold JM (2007). Overlooking the obvious: a meta-analytic comparison of digit symbol coding tasks and other cognitive measures in schizophrenia. Arch Gen Psychiatry.

[6] Addington J, Brooks BL, Addington D (2003). Cognitive functioning in first episode psychosis: initial presentation. Schizophrenia Res.

[7] Stone WS, Cai B, Liu X, Grivel MM, Yu G, Xu Y (2020). Association between the duration of untreated psychosis and selective cognitive performance in community-dwelling individuals with chronic untreated schizophrenia in rural China. JAMA Psychiatry.

[8] Stone WS, Phillips MR, Yang LH, Kegeles LS, Susser ES, Lieberman JA (2022). Neurodegenerative model of schizophrenia: Growing evidence to support a revisit. Schizophr Res.

[9] Cowman M, Holleran L, Lonergan E, O’Connor K, Birchwood M, Donohoe G (2021). Cognitive predictors of social and occupational functioning in early psychosis: a systematic review and meta-analysis of cross-sectional and longitudinal data. Schizophrenia Bull.

[10] Mucci A, Galderisi S, Gibertoni D, Rossi A, Rocca P, Bertolino A (2021). Factors associated with real-life functioning in persons with schizophrenia in a 4-year follow-up study of the italian network for research on psychoses. JAMA Psychiatry.

[11] Green MF, Kern RS, Heaton RK (2004). Longitudinal studies of cognition and functional outcome in schizophrenia: implications for MATRICS. Schizophrenia Res.

[12] Fervaha G, Foussias G, Agid O, Remington G (2014). Motivational and neurocognitive deficits are central to the prediction of longitudinal functional outcome in schizophrenia. Acta Psychiatr Scand.

[13] Santesteban-Echarri O, Paino M, Rice S, Gonzalez-Blanch C, McGorry P, Gleeson J (2017). Predictors of functional recovery in first-episode psychosis: a systematic review and meta-analysis of longitudinal studies. Clin Psychol Rev.

[14] Catalan A, Richter A, Salazar de Pablo G, Vaquerizo-Serrano J, Mancebo G, Pedruzo B (2021). Proportion and predictors of remission and recovery in first-episode psychosis: systematic review and meta-analysis. Eur Psychiatry.

[15] Fusar-Poli P, McGorry PD, Kane JM (2017). Improving outcomes of first-episode psychosis: an overview. World Psychiatry.

[16] Miley K, Hadidi N, Kaas M, Yu F (2020). Cognitive training and remediation in first-episode psychosis: a literature review. J Am Psychiatr Nurses Assoc.

[17] Lewandowski KE, Cohen BM, Oengur D (2011). Evolution of neuropsychological dysfunction during the course of schizophrenia and bipolar disorder. Psychological Med.

[18] Watson AJ, Harrison L, Preti A, Wykes T, Cella M (2022). Cognitive trajectories following onset of psychosis: a meta-analysis. Br J Psychiatry.

[19] Catalan A, Salazar de Pablo G, Aymerich C, Damiani S, Sordi V, Radua J (2021). Neurocognitive functioning in individuals at clinical high risk for psychosis a systematic review and meta-analysis. JAMA Psychiatry.

[20] Page MJ, McKenzie JE, Bossuyt PM, Boutron I, Hoffmann TC, Mulrow CD (2021). The PRISMA 2020 statement: an updated guideline for reporting systematic reviews. PLoS Med.

[21] Stroup DF, Berlin JA, Morton SC, Olkin I, Williamson GD, Rennie D (2000). Meta-analysis of observational studies in epidemiology: a proposal for reporting. Meta-analysis Of Observational Studies in Epidemiology (MOOSE) group. JAMA.

[22] Altman DG, Simera I, Hoey J, Moher D, Schulz K (2008). EQUATOR: reporting guidelines for health research. Lancet.

[23] Hauser M, Zhang J-P, Sheridan EM, Burdick KE, Mogil R, Kane JM (2017). Neuropsychological test performance to enhance identification of subjects at clinical high risk for psychosis and to be most promising for predictive algorithms for conversion to psychosis: a meta-analysis. J Clin Psychiatry.

[24] Nuechterlein KH, Green MF, Kern RS, Baade LE, Barch DM, Cohen JD (2008). The MATRICS Consensus Cognitive Battery, part 1: test selection, reliability, and validity. Am J Psychiatry.

[25] Kern RS, Nuechterlein KH, Green MF, Baade LE, Fenton WS, Gold JM (2008). The MATRICS Consensus Cognitive Battery, part 2: co-norming and standardization. Am J Psychiatry.

[26] McKenzie JE, Brennan SE, Ryan RE, Thomson HJ, Johnston RV. Chapter 9: Summarizing study characteristics and preparing for synthesis. In: Higgins JPT, Thomas J, Chandler J, Cumpston M, Li T, Page MJ, et al. editors. Cochrane Handbook for Systematic Reviews of Interventions version 6.4 (updated August 2023). Cochrane; 2023.

[27] Allott K, Wood SJ, Yuen HP, Yung AR, Nelson B, Brewer WJ (2019). Longitudinal cognitive performance in individuals at ultrahigh risk for psychosis: a 10-year follow-up. Schizophrenia Bull.

[28] Hedges EP, See C, Si S, McGuire P, Dickson H, Kempton MJ (2022). Meta-analysis of longitudinal neurocognitive performance in people at clinical high-risk for psychosis. Psychol Med.

[29] Maslej MM, Furukawa TA, Cipriani A, Andrews PW, Sanches M, Tomlinson A (2021). Individual differences in response to antidepressants: a meta-analysis of placebo-controlled randomized clinical trials. JAMA Psychiatry.

[30] Nakagawa S, Poulin R, Mengersen K, Reinhold K, Engqvist L, Lagisz M (2015). Meta-analysis of variation: ecological and evolutionary applications and beyond. Methods Ecol Evol.

[31] Ploderl M, Hengartner MP (2019). What are the chances for personalised treatment with antidepressants? Detection of patient-by-treatment interaction with a variance ratio meta-analysis. BMJ Open.

[32] DerSimonian R, Laird N (1986). Meta-analysis in clinical trials. Control Clin Trials.

[33] Lipsey MW, Wilson DB. Practical meta-analysis. Applied Social Research Methods. Vol 49, Sage Publications; 2000.

[34] Duval S, Tweedie R (2000). Trim and fill: A simple funnel-plot-based method of testing and adjusting for publication bias in meta-analysis. Biometrics.

[35] R Foundation for Statistical Computing. R: a language and environment for statistical computing. 1.4.1106 ed. Vienna, Austria: R Foundation for Statistical Computing; 2021.

[36] The Comprehensive R Archive Network. Package ‘Metafor’. 2015, https://cran.r-project.org/web/packages/metafor/index.html.

[37] Tan EJ, Rossell SL, Subotnik KL, Ventura J, Nuechterlein KH (2021). Cognitive heterogeneity in first-episode psychosis and its relationship with premorbid developmental adjustment. Psychol Med.

[38] Reser MP, Allott KA, Killackey E, Farhall J, Cotton SM (2015). Exploring cognitive heterogeneity in first-episode psychosis: what cluster analysis can reveal. Psychiatry Res.

[39] Murray RM, Bora E, Modinos G, Vernon A (2022). Schizophrenia: a developmental disorder with a risk of non-specific but avoidable decline. Schizophr Res.

[40] Fett A-KJ, Velthorst E, Reichenberg A, Ruggero CJ, Callahan JL, Fochtmann LJ (2020). Long-term changes in cognitive functioning in individuals with psychotic disorders findings from the suffolk county mental health project. JAMA Psychiatry.

[41] Calamia M, Markon K, Tranel D (2012). Scoring higher the second time around: meta-analyses of practice effects in neuropsychological assessment. Clin Neuropsychologist.

[42] Basso MR, Lowery N, Ghormley C, Bornstein RA (2001). Practice effects on the Wisconsin Card Sorting Test-64 Card version across 12 months. Clin Neuropsychol.

[43] Beglinger LJ, Gaydos B, Tangphao-Daniels O, Duff K, Kareken DA, Crawford J (2005). Practice effects and the use of alternate forms in serial neuropsychological testing. Arch Clin Neuropsychol.

[44] Basso MR, Bornstein RA, Lang JM (1999). Practice effects on commonly used measures of executive function across twelve months. Clin Neuropsychol.

[45] Diamond A, Silverstein SM, Keane BP (2022). Visual system assessment for predicting a transition to psychosis. Transl Psychiatry.

[46] van Loon AM, Fahrenfort JJ, van der Velde B, Lirk PB, Vulink NCC, Hollmann MW (2016). NMDA receptor antagonist ketamine distorts object recognition by reducing feedback to early visual cortex. Cereb Cortex.

[47] Abram SV, Roach BJ, Fryer SL, Calhoun VD, Preda A, van Erp TGM (2022). Validation of ketamine as a pharmacological model of thalamic dysconnectivity across the illness course of schizophrenia. Mol Psychiatry.

[48] Burton BK, Krantz MF, Skovgaard LT, Brandt JM, Gregersen M, Sondergaard A (2023). Impaired motor development in children with familial high risk of schizophrenia or bipolar disorder and the association with psychotic experiences: a 4-year Danish observational follow-up study. Lancet Psychiatry.

[49] Bernard JA, Mittal VA (2015). Updating the research domain criteria: the utility of a motor dimension. Psychol Med.

[50] Ali T, Sisay M, Tariku M, Mekuria AN, Desalew A (2021). Antipsychotic-induced extrapyramidal side effects: a systematic review and meta-analysis of observational studies. PLoS One.

[51] Keefe RS, Bilder RM, Davis SM, Harvey PD, Palmer BW, Gold JM (2007). Neurocognitive effects of antipsychotic medications in patients with chronic schizophrenia in the CATIE Trial. Arch Gen Psychiatry.

[52] Ayesa-Arriola R, Rodríguez-Sánchez JM, Pérez-Iglesias R, Roiz-Santiáñez R, Martínez-García O, Sánchez-Moreno J (2013). Long-term (3-year) neurocognitive effectiveness of antipsychotic medications in first-episode non-affective psychosis: a randomized comparison of haloperidol, olanzapine, and risperidone. Psychopharmacology.

[53] Birnbaum ML, Wan CR, Broussard B, Compton MT (2017). Associations between duration of untreated psychosis and domains of positive and negative symptoms. Early Inter Psychiatry.

[54] Green MF, Marshall BD, Wirshing WC, Ames D, Marder SR, McGurk S (1997). Does risperidone improve verbal working memory in treatment-resistant schizophrenia?. Am J Psychiatry.

[55] Gonzalez-Ortega I, de los Mozos V, Echeburua E, Mezo M, Besga A, Ruiz de Azua S (2013). Working memory as a predictor of negative symptoms and functional outcome in first episode psychosis. Psychiatry Res.

[56] Haatveit B, Vaskinn A, Sundet KS, Jensen J, Andreassen OA, Melle I (2015). Stability of executive functions in first episode psychosis: One year follow up study. Psychiatry Res.

[57] Fusar-Poli P, Cappucciati M, Rutigliano G, Heslin M, Stahl D, Brittenden Z (2016). Diagnostic stability of ICD/DSM first episode psychosis diagnoses: meta-analysis. Schizophr Bull.

