# Supplementary material for: The magnitude and variability of neurocognitive performance in first-episode psychosis: a systematic review and meta-analysis of longitudinal studies
Source: Transl Psychiatry. 2024 Jan 8;14:15. doi: 10.1038/s41398-023-02718-6 (PMC10774360; doi:10.1038/s41398-023-02718-6)
Supplement: Supplementary file 1 — supplementary material [file 41398_2023_2718_MOESM1_ESM.docx]

Supplementary material

[eTable 1. PRISMA statement and checklist. 2](#_Toc130376065)

[eTable 2. MOOSE checklist 4](#_Toc130376066)

[eMethods 1. Search terms 6](#_Toc130376067)

[eMethods 2. Methods for pooling non-independent cognitive tasks 6](#_Toc130376068)

[eMethods 3. Methods for analyzing variability 6](#_Toc130376069)

[eTable 3. Cognitive domains considered in the current meta-analysis (7 MATRICS domains and 8 extra domains) 8](#_Toc130376070)

[eTable 4. Risk of bias (quality) assessment using modified Newcastle-Ottawa Scale for cross-sectional and cohort studies 9](#_Toc130376071)

[eTable 5. Characteristics of included studies 10](#_Toc130376072)

[eTable 6. Variability between FEP and HC at baseline and follow-up 15](#_Toc130376073)

[eFigure 1. Comparison between FEP and HC at baseline 17](#_Toc130376074)

[eTable 7. Meta-analytical comparisons: FEP vs HC at baseline 18](#_Toc130376075)

[eTable 8. Meta-analytical comparisons: FEP vs HC at follow-up. 20](#_Toc130376076)

[eFigure 2. Comparison between FEP and HC at follow-up 21](#_Toc130376077)

[eTable 9. Comparisons between FEP and HC domains merged 22](#_Toc130376078)

[eTable 10. Metaregressions FEP vs HC 23](#_Toc130376079)

[eTable 11. Meta-analytical comparisons: FEP baseline vs FEP at follow-up. 26](#_Toc130376080)

[eFigure 3. Comparisons between FEP at baseline vs follow-up domains merged 29](#_Toc130376081)

[eTable 12. Metaregressions FEP baseline vs follow-up 30](#_Toc130376082)

[eFigure 4. Funnel plots 34](#_Toc130376083)

# **eTable 1. PRISMA statement and checklist.**

| **Section and Topic** | **Item #** | **Checklist item** | **Location where item is reported** |
| --- | --- | --- | --- |
| **TITLE** | | |  |
| Title | 1 | Identify the report as a systematic review. | Cover page |
| **ABSTRACT** | | |  |
| Abstract | 2 | See the PRISMA 2020 for Abstracts checklist. | Abstract |
| **INTRODUCTION** | | |  |
| Rationale | 3 | Describe the rationale for the review in the context of existing knowledge. | Introduction |
| Objectives | 4 | Provide an explicit statement of the objective(s) or question(s) the review addresses. | Introduction |
| **METHODS** | | |  |
| Eligibility criteria | 5 | Specify the inclusion and exclusion criteria for the review and how studies were grouped for the syntheses. | Methods |
| Information sources | 6 | Specify all databases. registers. websites. organisations. reference lists and other sources searched or consulted to identify studies. Specify the date when each source was last searched or consulted. | Methods |
| Search strategy | 7 | Present the full search strategies for all databases. registers and websites. including any filters and limits used. | Methods |
| Selection process | 8 | Specify the methods used to decide whether a study met the inclusion criteria of the review. including how many reviewers screened each record and each report retrieved. whether they worked independently. and if applicable. details of automation tools used in the process. | Methods |
| Data collection process | 9 | Specify the methods used to collect data from reports. including how many reviewers collected data from each report. whether they worked independently. any processes for obtaining or confirming data from study investigators. and if applicable. details of automation tools used in the process. | Methods |
| Data items | 10a | List and define all outcomes for which data were sought. Specify whether all results that were compatible with each outcome domain in each study were sought (e.g. for all measures. time points. analyses). and if not. the methods used to decide which results to collect. | Methods |
|  | 10b | List and define all other variables for which data were sought (e.g. participant and intervention characteristics. funding sources). Describe any assumptions made about any missing or unclear information. | Methods |
| Study risk of bias assessment | 11 | Specify the methods used to assess risk of bias in the included studies. including details of the tool(s) used. how many reviewers assessed each study and whether they worked independently. and if applicable. details of automation tools used in the process. | Methods |
| Effect measures | 12 | Specify for each outcome the effect measure(s) (e.g. risk ratio. mean difference) used in the synthesis or presentation of results. | Methods |
| Synthesis methods | 13a | Describe the processes used to decide which studies were eligible for each synthesis (e.g. tabulating the study intervention characteristics and comparing against the planned groups for each synthesis (item #5)). | Methods |
|  | 13b | Describe any methods required to prepare the data for presentation or synthesis. such as handling of missing summary statistics. or data conversions. | Methods |
|  | 13c | Describe any methods used to tabulate or visually display results of individual studies and syntheses. | Methods |
|  | 13d | Describe any methods used to synthesize results and provide a rationale for the choice(s). If meta-analysis was performed. describe the model(s). method(s) to identify the presence and extent of statistical heterogeneity. and software package(s) used. | Methods |
|  | 13e | Describe any methods used to explore possible causes of heterogeneity among study results (e.g. subgroup analysis. meta-regression). | Methods |
|  | 13f | Describe any sensitivity analyses conducted to assess robustness of the synthesized results. | Methods |
| Reporting bias assessment | 14 | Describe any methods used to assess risk of bias due to missing results in a synthesis (arising from reporting biases). | Methods |
| Certainty assessment | 15 | Describe any methods used to assess certainty (or confidence) in the body of evidence for an outcome. | Methods |
| **RESULTS** | | |  |
| Study selection | 16a | Describe the results of the search and selection process. from the number of records identified in the search to the number of studies included in the review. ideally using a flow diagram. | Results |
|  | 16b | Cite studies that might appear to meet the inclusion criteria. but which were excluded. and explain why they were excluded. | Results |
| Study characteristics | 17 | Cite each included study and present its characteristics. | Results |
| Risk of bias in studies | 18 | Present assessments of risk of bias for each included study. | Results |
| Results of individual studies | 19 | For all outcomes. present. for each study: (a) summary statistics for each group (where appropriate) and (b) an effect estimates and its precision (e.g. confidence/credible interval). ideally using structured tables or plots. | Results |
| Results of syntheses | 20a | For each synthesis. briefly summarise the characteristics and risk of bias among contributing studies. | Results |
|  | 20b | Present results of all statistical syntheses conducted. If meta-analysis was done. present for each the summary estimate and its precision (e.g. confidence/credible interval) and measures of statistical heterogeneity. If comparing groups. describe the direction of the effect. | Results |
|  | 20c | Present results of all investigations of possible causes of heterogeneity among study results. | Results |
|  | 20d | Present results of all sensitivity analyses conducted to assess the robustness of the synthesized results. | Results |
| Reporting biases | 21 | Present assessments of risk of bias due to missing results (arising from reporting biases) for each synthesis assessed. | Results |
| Certainty of evidence | 22 | Present assessments of certainty (or confidence) in the body of evidence for each outcome assessed. | Results |
| **DISCUSSION** | | |  |
| Discussion | 23a | Provide a general interpretation of the results in the context of other evidence. | Discussion |
|  | 23b | Discuss any limitations of the evidence included in the review. | Discussion |
|  | 23c | Discuss any limitations of the review processes used. | Discussion |
|  | 23d | Discuss implications of the results for practice. policy. and future research. | Discussion |
| **OTHER INFORMATION** | | |  |
| Registration and protocol | 24a | Provide registration information for the review. including register name and registration number. or state that the review was not registered. | <https://osf.io/r94t5/> |
|  | 24b | Indicate where the review protocol can be accessed. or state that a protocol was not prepared. | OSF Home |
|  | 24c | Describe and explain any amendments to information provided at registration or in the protocol. |  |
| Support | 25 | Describe sources of financial or non-financial support for the review. and the role of the funders or sponsors in the review. | No funders |
| Competing interests | 26 | Declare any competing interests of review authors. | No competing interests |
| Availability of data. code and other materials | 27 | Report which of the following are publicly available and where they can be found: template data collection forms; data extracted from included studies; data used for all analyses; analytic code; any other materials used in the review. | Data accessible upon request |

# **eTable 2. MOOSE checklist**

| **Criteria** | | **Brief description of how the criteria were handled in the meta-analysis** |
| --- | --- | --- |
| **Reporting of background should include** | |  |
| √ | Problem definition | To examine at a meta-analytical level whether neurocognitive deficits are evident in First Episode Psychosis patients (FEP) relative to healthy controls (HC) and to define the specific pattern of these neurocognitive deficits.  To identify neurocognitive impairments evolution that specifically cuured the later transition to psychosis in the FEP population. controlling for the potential confounding effect of socio-demographical. methodological. and clinical factors. |
| √ | Hypothesis statement | We hypothesized that FEP group would have a significant impairment in neurocognitive domains. and a higher impairment at the follow-up. |
| √ | Description of study outcomes | In line with our earlier meta-analysis the different neurocognitive tasks were grouped in neurocognitive domains on the basis of the criteria developed by the MATRICS conference and then discussed by us. according to the indications of the articles included: (1) processing speed. (2) verbal learning. (3) working memory. (4) reasoning and problem-solving. (5) visual learning. (5) attention and vigilance. and (7) social cognition. Further. we have analysed the CHR-P domains of (8) general intelligence. (9) premorbid intelligence. (10) visuospatial ability. (11) verbal memory. (12) visual memory. (13) executive functioning. (14) motor functioning. and (15) olfaction. We reported differences between FEP population and HC in these domains. measured by standardised scales.  For comprehensiveness. we conducted two supplementary meta-analyses: (i) estimating the pooled effect sizes across each of the neurocognitive domains. For the latter meta-analysis (ii). we followed meta-analytical guidelines^1,2^ to account for studies reporting on more than one non-independent neurocognitive tasks within the same neurocognitive domain. |
| √ | Type of exposure or intervention used | We included individual longitudinal studies that reported neurocognitive data in FEP population. |
| √ | Type of study designs used | Case-control studies. and cohort studies. which investigate the neurocognitive functioning in FEP compared to HC. |
| √ | Study population | FEP individuals. |
| **Reporting of search strategy should include** | |  |
| √ | Qualifications of researchers | The credentials of the investigators are indicated in the author list and in the acknowledgements. |
| √ | Search strategy. including time period included in the synthesis and keywords | We performed a multi-step literature search using the following keywords: "“longitudinal” OR “follow-up” AND "cognit*" OR "neurocognit*" OR "social cognit*" OR “neuropsy*” AND "psychosis risk" OR "prodrom*" OR "ultra-high risk" OR "clinical high risk" OR "genetic high risk" OR "at risk mental state" OR "at-risk mental state" OR "basic symptoms" OR “bipolar disorder” OR “manic” OR “first episode psychosis” OR “psychosis” OR “schizophrenia” OR “bipo*”OR “depress*” from inception until 1^st^ November 2022. |
| √ | Databases and registries searched | Web of Science database (Clarivate Analytics): Web of Science Core Collection. BIOSIS Citation Index. KCI-Korean Journal Database. MEDLINE. Russian Science Citation Index. PubMed and SciELO Citation Index. |
| √ | Use of hand searching | We hand-searched bibliographies of retrieved papers for additional references. |
| √ | List of citations located and those excluded. including justifications | Details of the literature search process are outlined in the results section and in the PRISMA flow-chart. |
| √ | Method of addressing articles published in languages other than English | Only articles in English language were selected. |
| √ | Method of handling abstracts and unpublished studies | Original individual studies were included. Conference proceedings. reviews. editorials. clinical cases and unpublished studies were excluded. |
| √ | Description of any contact with authors | No attempt was made to contact the corresponding authors to request additional data for this study. |
| **Reporting of methods should include** | |  |
| √ | Description of relevance or appropriateness of studies assembled for assessing the hypothesis to be tested | Detailed inclusion and exclusion criteria were described in the methods section. |
| √ | Rationale for the selection and coding of data | Data extracted from each of the studies were relevant to the population characteristics. study design. comparison group. exposure and outcomes. |
| √ | Assessment of confounding factors | Confounding factors were systematically assessed in each neurocognitive domain. |
| √ | Assessment of study quality | We adapted the Newcastle-Ottawa Scale for the evaluation of cross-sectional and cohort studies. |
| √ | Assessment of heterogeneity | Heterogeneity was assessed with the I^2^ index. |
| √ | Description of statistical methods in sufficient detail to be replicated | Statistical methods are described in detail in the methods section. |
| √ | Provision of appropriate tables and graphics | We included the PRISMA flow-chart and several tables and graphics to describe the literature search and our results. |
| **Reporting of results should include** | |  |
| √ | Graph summarizing individual study estimates and overall estimate | We have appended them in the main text. Additional graphs were presented as supplementary material to fully describe the results. |
| √ | Table giving descriptive information for each study included | We have presented descriptive information for each study in the supplementary material. |
| √ | Results of sensitivity testing | Subgroup analyses were conducted to analyse differences between used task in each neurocognitive domain. |
| √ | Indication of statistical uncertainty of findings | We reported mean estimates for the main outcome and 95% CI. |
| **Reporting of discussion should include** | |  |
| √ | Quantitative assessment of bias | Publication biases were assessed by funnel plots visual inspections and trim and fill test^3^. The trim and fill methods were used as sensitivity analyses to correct biases if detected. |
| √ | Justification for exclusion | Exclusion criteria and justification are described in the manuscript. |
| √ | Assessment of quality of included studies | We adapted the Newcastle-Ottawa Scale for the evaluation of cross-sectional and cohort studies. |
| **Reporting of conclusions should include** | |  |
| √ | Consideration of alternative explanations for observed results | We discussed other explanations for our findings in the discussion section. |
| √ | Generalization of the conclusions | We have addressed the generalization of the conclusions in the discussion section. |
| √ | Guidelines for future research | We have suggested possible streams of future development and research in the discussion. |
| √ | Disclosure of funding source | Funding source described at the end of the manuscript. No separate funding was necessary for the undertaking of this meta-analysis. |

# **eMethods 1. Search terms**

The following search terms in various combinations were applied: “longitudinal” OR “follow-up” AND "cognit*" OR "neurocognit*" OR "social cognit*" OR “neuropsy*” AND "psychosis risk" OR "prodrom*" OR "ultra-high risk" OR "clinical high risk" OR "genetic high risk" OR "at risk mental state" OR "at-risk mental state" OR "basic symptoms" OR “bipolar disorder” OR “manic” OR “first episode psychosis” OR “psychosis” OR “schizophrenia” OR “bipo*” OR “depress*”.

# **eMethods 2. Methods for pooling non-independent cognitive tasks**

To estimate the pooled effect size in case of studies reporting more than one non-independent cognitive task within the same cognitive domains. we assumed a correlation of 0.3 ^4-6^ between the non-independent tasks. However. to ensure that the results did not depend on this assumption. we also conducted the meta-analysis. assuming that the correlation was either 0.1 or 0.5. We first computed the variance of the average effect size. then we multiplied it by (1+(n.es-1) * r)/n.es. where n.es is the number of combined effect sizes and r the correlation between non-independent cognitive tasks.

# **eMethods 3. Methods for analyzing variability**

For each observation corresponding to a group (FEP or HC). we calculated the log SD:


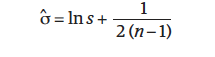


where s refers to the SD and n refers to the number of participants for that group. We used the following formula to derive sampling variances:


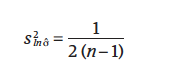


where n refers to the number of participants for that group.

In our primary RSMM. we specified each ln σˆ (fitted with its associated sampling variance) as the response variable. To account for the mean-SD association. we added the log end point mean score (z-transformed across the entire data set) as a predictor. Because the effect of treatment group. estimated by bln σˆ. represented the difference in ln σˆ between antidepressant and placebo.18 we report the exponentiated bln σˆ. A group effect larger than 1 indicated higher variability in antidepressant groups than placebo groups; conversely. a group effect lower than 1 indicated less variability in the antidepressant groups compared with placebo groups.

# **eTable 3.** **Cognitive domains considered in the current meta-analysis (7 MATRICS domains and 8 extra domains)**

Adapted from Fusar-Poli et al. 2012 ^7^ and Hauser et al. 2017^8^

| **Cognitive domains** | **Tasks** |
| --- | --- |
| ***MATRICS domains*** | |
| **Processing Speed** | - Trail Making Test-Part A (TMT-A)^9^ - Basic Assessment of Cognition Scale (BACS)^10^ - Controlled Oral Word Association Test (COWAT)^11^ - Category fluency^12^ - Animal fluency^13^ - Letter fluency^14^ - Stroop Word Test (Stroop W)^15^ - Stroop Colour Test (Stroop C)^15^ - Stroop Colour-Word Test (Stroop C-W)^15^ |
| **Attention/Vigilance** | - Continuous Performance Test – Identical Pairs (CPT-IP)*^16,17^ and reaction time |
| **Working Memory** | - Wechsler Memory Scale. 3^rd^ ed. Spatial Span Subtest (WMS-III: SS)^18^ - Letter Number Span (LNS)^19^ - Letter Number Sequencing Test (LNST)^20^ - WAIS digit backwards^21^ - WAIS digit forwards^21^ |
| **Verbal Learning** | - Hopkins Verbal Learning Test—Revised (HVLT-R)**^22^ - Rey Auditory Verbal Learning Test (RAVLT)***^23^ - California Verbal Learning Test I (CVLT)^23-25^ - Logical memory immediate recall (LM immediate recall)^21^ - WMS- paired associated^21^ |
| **Visual learning** | - Brief Visuospatial Memory Test Revised (BVMT-R)**^26^ - Wechsler Memory Scale Visual Memory immediate recall (WMS VM)^18^ - Rey–Osterrieth Complex Figure test Immediate Recall (ROCF)^27^ - Benton test: number errors. number corrects^28^ |
| **Reasoning and Problem Solving** | - Neuropsychological Assessment Battery Mazes (NAB Mazes)^29^ |
| **Social cognition** | - Mayer-Salovey-Caruso Emotional Intelligence Test (MSCEIT)^30^ |
| ***Extra domains*** | |
| **General intelligence IQ** | - Wechsler Adult Intelligence Scale. 3^rd^ edition (WAIS III)^18^ - Wechsler Adult Intelligence Scale-Revised (WAIS-R)^31^ - Wechsler Adult Intelligence Scale -IV (WAIS IV)^21^ |
| **Visuospatial ability** | - WAIS/WISC Block Design (WAIS/WISC BD)^20,32^ |
| **Verbal memory** | - RAVLT delayed recall (RAVLT DR)^23^ - CVLT delayed recall (CVLT DR)^23-25^ - WMS logical memory delayed recall^17^ |
| **Executive functioning** | - Trail Making Test- Part B (TMT- B)^9^ - Wisconsin Card Sorting Test (WCST)^33^: categories. number of errors. perseverative errors and perseverative responses - Stroop interference^34^ |
| **Motor functioning** | - Finger Tapping Test (Tapping)^35^ - Grooved Pegboard Test (GPT)^36^ |

*****Mean d' across conditions; **Total Learning Trials 1-5; ***Learning Trials

# **eTable 4.** Risk of bias (quality) assessment using modified Newcastle-Ottawa Scale for cross-sectional and cohort studies

| **Newcastle-Ottawa Scale Criteria** | **Maximum Score** |
| --- | --- |
| *Cross-Sectional Studies* | |
| Sample representative of target sample (e.g. all eligible or random sample)? | 2 |
| Sample size justified and satisfactory? | 1 |
| Non-response rate is defined satisfactory. and characteristics of responders/non-responders compared? | 1 |
| Ascertainment of exposure (i.e. menstrual cycle) is valid and/or well described? | 1 |
| Assessment of outcome with robust tool and/or record linkage? | 2 |
| Outcome per group reported appropriately? | 1 |
| *Cohort Studies* | |
| Representativeness of exposed cohort (e.g. total population or random sample. selected group) | 1 |
| Method used to ascertain exposure (menstrual cycle phase) is robust? | 1 |
| Exposed and unexposed are matched or adjustment for confounding factors? | 2 |
| Assessment of outcome was blind to exposure status or used record linkage. were robust tools used? | 2 |
| Follow-up period was sufficiently long for outcomes to occur (e.g. more than one menstrual cycle? | 1 |
| Loss to follow-up rate is reported. low (<30%). and same in exposed and non-exposed? | 1 |

# **eTable 5**. Characteristics of included studies

FEP. First Episode Psychosis; HC. Healthy Controls; FUP. Follow-up; mo. months; NOS. Newcastle-Ottawa Scale.

| *Author year* | *Country* | *N FEP* | *N HC* | *Age mean (SD)* | *FUP (mo)* | *Task used* | *NOS* |
| --- | --- | --- | --- | --- | --- | --- | --- |
| Addington2005^37^ | Canada | 247 | 66 | 24.12 (8.05) | 24 | COWAT; Grooved Pegboard Test; RVLT-delayed recall; ROCFT copy; RAVLT 1-5;  RVLT long delay free recall; ROCF delayed recall; Stroop-word; TMT-A; TMT-B; WMS-R-LM II; WMS-R-LM I;  WMS-LM immediate recall;  WMS- LM delayed recall;  WCST categories; WCST perseverative errors | 8 |
| Albus2002^38^ | Germany | 50 | 50 | 30.3 (9.54) | 24 | CVLT immediate recall; TMT-B; WCST categories; TMT-B; WAIS digit symbol; WCST-perseverative responses; WMS-R-LM II; WMS-R-LM I; WMS-LM immediate recall; WMS- LM delayed recall;  WMS-Paired associated;  WMS Visual Memory immediate recall;  WMS delayed recall | 7 |
| Ayesa-Arriola2013^39^ | Spain | 79 | 41 | 27.62 (7.95) | 36 | RAVLT 1-5 | 7 |
| Ayres2010^40^ | Brasil | 56 | 70 | 29.36 (8.50) | >36 | COWAT; Digit span forward; WAIS digit backwards; WAIS digit forwards | 5 |
| Barder2013^41^ | Norway | 62 | 0 | 28.00 (9.00) | 60 | CPT-reaction time; CVLT 1-5; Tapping test; WCST categories | 6 |
| Barder2014^42^ | Norway | 43 | 0 | 28.00 (9.00) | 120 | Tapping test | 5 |
| Burdick2006^43^ | USA | 16 | 16 | 37.85 (5.32) | 60 | CVLT immediate recall; CVLT 1-5; TMT-A; TMT-B; WCST-perseverative errors; | 6 |
| Chan2014^44^ | China | 92 | 0 | 26.6 (11.20) | 12 | WCST categories; WMS-LM immediate recall; | 6 |
| Chan2015^45^ | China | 101 | 0 | 24.51 (6.23) | 6 | WCST categories; WMS- LM delayed recall; WMS Visual Memory immediate recall; WMS delayed recall | 6 |
| Chang2014^46^ | China | 93 | 0 | 31.20 (9.60) | 36 | Digit span forward; WCST perseverative errors; WMS Visual Memory immediate recall; | 6 |
| Crespo-Facorro2009^47^ | Spain | 79 | 41 | 26.63 (6.42) | 60 | Tapping test; RVLT long delay free recall | 6 |
| Cuesta2014^48^ | Spain | 77 | 0 | 30.08 (10.90) | 6 | Animal fluency; TMT-B; WCST perseverative errors; WMS-Paired associated; WMS Visual Memory immediate recall | 6 |
| Faber2012^49^ | Netherlands | 42 | 0 | 25.60 (n.a) | 5 | Animal fluency; Category fluency; CPT-reaction time; Stroop colour; Stroop colour-word; TMT-A; TMT-B; WAIS digit backwards; WAIS digit forwards; | 7 |
| Fett2020^50^ | USA | 211 | 0 | 28.51 (8.50) | 240 | COWAT; TMT-A; TMT-B; Stroop colour-word; WAIS digit symbol; WMS Visual Memory immediate recall; WMS delayed recall | 8 |
| Frangou2008^51^ | UK | 20 | 20 | 15.78 (1.70) | 48 | Stroop-word; IQ total; IQ verbal; IQ performance; TMT-A; TMT-B; | 8 |
| Gold1999^52^ | USA | 54 | 0 | 24.00 (4.73) | 60 | IQ total; IQ verbal; IQ performance; Tapping test; TMT-B; ROCF delayed recall; WCST categories; WMS-LM immediate recall | 6 |
| Gonzalez-Blanch2006^53^ | Spain | 42 | 43/43 | 26.00 (6.85) | 3 | LNST | 8 |
| Gonzalez-Ortega2013^54^ | Spain | 26 | 0 | 25.77 (7.46) | 54 | Animal fluency; Digit span forward; LNST; Stroop colour; Stroop interference; TMT-A; TMT-B; WCST perseverative errors; WCST number errors; WMS-Paired associated; WMS- LM delayed recall; WAIS digit backwards | 6 |
| Haatveit2015^55^ | Norway | 82 | 107 | 27.78 (7.22) | 12 | Category fluency; Letter fluency; LNST; WCST-perseverative responses; WAIS digit backwards | 8 |
| Hallgren2019^56^ | Sweden | 91 | 0 | 30.00 (7.3) | 3 | TMT-A; TMT-B | 6 |
| Harvey2005^57^ | Multiple sites | 338 | 0 | 25.47(6.93) | 3 | Animal fluency; Letter fluency; CPT-IP d' total score; RAVLT 1-5; RVLT long delay free recall;  WCST categories; WCST perseverative errors; WMS Visual Memory immediate recall; WMS delayed recall | 8 |
| Hill2004^58^ | USA | 45 | 33 | 25.00 (7.17) | 24 | CVLT immediate recall;  CVLT long delay free recall;  COWAT; CVLT 1-5; Digit span forward; Stroop-word; Grooved Pegboard Test; Tapping test; TMT-A; TMT-B; WAIS digit symbol; WCST perseverative errors; WMS Visual Memory immediate recall; WMS delayed recall | 6 |
| Hoff2005^59^ | USA | 21 | 8 | 38.31 (5.77) | 120 | Benton; CVLT 1-5; Stroop-word; IQ total; IQ verbal; Tapping test; TMT-A; TMT-B; WCST perseverative errors; WCST number errors; WMS-LM immediate recall; WMS- LM delayed recall; WMS Visual Memory immediate recall; WMS delayed recall | 4 |
| Horan2012^60^ | USA | 55 | 0 | 22.30 (4.30) | 12 | MSCEIT | 6 |
| Jahshan2010^61^ | USA | 20 | 29 | 19.45 (5.54) | 6 | HVLT total recall; LNST; Stroop colour; Stroop interference; WCST-perseverative responses; WAIS Block Design; WMS-III: Spatial Span | 6 |
| Kenney2015^62^ | Ireland | 37 | 59 | 28.73 (7.71) | 48 | HVLT total recall; BVMT-R; MSCEIT; NAB Mazes | 7 |
| Kopala2006^63^ | Canada | 20 | 0 | 23.20 (6.10) | 24 | Benton; Grooved Pegboard Test; Letter fluency; Tapping test; TMT-A; TMT-B; CPT-IP d' total score; WCST perseverative errors; | 5 |
| Kuharic2021^64^ | Croatia | 129 | 100 | 25.33 (7.46) | 18 | RAVLT 1-5; RVLT long delay free recall;  Stroop colour; Stroop word; Stroop colour-word; TMT-A; ROCF delayed recall; WMS-Paired associated; WAIS Block Design; WAIS digit backwards; WAIS digit forwards | 7 |
| Labad2016^65^ | Spain | 36 | 50 | 24.22 (5.02) | 12 | Animal fluency; BAC symbol coding; BVMT-R HVLT total recall; LNS; NAB Mazes; Stroop-word; TMT-A; | 8 |
| Leeson2009^66^ | UK | 54 | 0 | n.a | 52 | IQ total; IQ verbal; | 5 |
| Malla2002^67^ | Canada | 66 | 0 | 25.60 (8.90) | 12 | IQ total; | 6 |
| Mayoral2008^68^ | Spain | 22 | 25 | 15.55 (1.64) | 24 | Category fluency; COWAT; CVLT 1-5; CVLT immediate recall; CVLT long delay free recall;  LNST; Stroop-word; TMT-A; TMT-B; WAIS digit backwards; WAIS digit forwards; WCST categories; WCST perseverative errors; | 7 |
| Minor2015^69^ | USA | 47 | 0 | 22.00 (3.50) | 6 | BAC symbol coding; BVMT-R CPT-IP d' total score; HVLT total recall; LNS; NAB Mazes; TMT-A; WMS-III: Spatial Span; | 6 |
| Mohn2018^70^ | Norway | 56 | 25 | 21.00 (2.63) | 24 | BVMT-R; CPT-reaction time; HVLT total recall | 8 |
| Molina2014^71^ | Spain | 31 | 23 | 24.98 (5.12) | 48 | WCST categories; RVLT long delay free recall;  ROCF delayed recall | 7 |
| Nopoulos1994^72^ | USA | 35 | 68 | 23.71 (4.55) | 24 | Benton; COWAT; RAVLT 1-5; Stroop colour; Stroop interference; WMS- LM delayed recall | 6 |
| Olivier2015^73^ | South Africa | 92 | 100 | 25.00 (6.80) | 12 | BVMT-R; NAB Mazes | 7 |
| Peña2011^74^ | Spain | 86 | 34 | 28.50 (7.50) | 24 | LNST; Stroop colour; Stroop word; TMT-A; WAIS digit symbol; WAIS digit backwards; WCST categories; WCST perseverative errors; WMS-LM immediate recall | 6 |
| Rodriguez-Sanchez2020^75^ | Spain | 140 | 40 | 29.27 (8.38) | 12 | Grooved Pegboard Test; ROCF delayed recall; WAIS digit symbol; | 7 |
| Rund2007^76^ | Norway | 301 | 0 | 28.20 (9.00) | 24 | COWAT; CVLT immediate recall; CVLT long delay free recall; TMT-A; TMT-B; CPT-IP d' total score; CPT-reaction time; WCST categories; WCST perseverative errors; WCST-perseverative responses | 6 |
| Saeedi2007^77^ | Canada | 190 | 0 | 24.40 (8.00) | 36 | Category fluency; Grooved Pegboard Test; Stroop colour-word; ROCFT copy; ROCF delayed recall; WMS- LM delayed recall | 6 |
| Sánchez-Torres2018^78^ | Spain | 159 | 151 | 26.42 (5.96) | 24 | Animal fluency; CPT-IP d' total score; CVLT immediate recall; CVLT long delay free recall; LNST; MSCEIT; Stroop colour-word; Stroop-word; TMT-A; TMT-B; WCST categories; WCST perseverative errors; WCST number errors | 7 |
| Setien-Suero2017^79^ | Spain | 311 | 0 | 27.41 (8.10) | 36 | WAIS digit backwards | 6 |
| Setien-Suero2021^80^ | Spain | 115 | 0 | 31.69 (10.34) | 36 | CPT-reaction time; RAVLT 1-5; Stroop colour-word; TMT-A; ROCFT copy | 6 |
| Stirling2003^81^ | UK | 37 | 0 | 26.30 (n.a) | >120 | COWAT; WCST categories; WAIS Block Design | 6 |
| Singh2022^82^ | India | 71 | 0 | 34.44 (7.17) | 6 | Letter fluency. category fluency | 6 |
| SSRG1988^83^ | UK | 28 | 0 | n.a | 12 | WAIS Block Design | 6 |
| Torgalsboen2014^84^ | Norway | 28 | 28 | 21.00 (2.60) | 6 | HVLT total recall | 7 |
| Torgalsboen2015^85^ | Norway | 25 | 25 | 21.00 (2.60) | 24 | NAB Mazes; MSCEIT | 7 |
| Torrent2018^86^ | Spain | 142 | 0 | 23.52 (5.76) | 24 | COWAT; CVLT 1-5; Digit span forward; Stroop interference | 7 |
| Townsend2002^87^ | Canada | 83 | 0 | 24.90 (7.90) | 12 | CPT-IP d' total score; IQ performance; Letter fluency; Stroop colour-word; TMT-A; TMT-B; Stroop colour; WCST categories; WCST perseverative errors; WMS Visual Memory immediate recall | 6 |
| Trampush2015^88^ | USA | 125 | 0 | 22.55 (5.67) | 3 | Animal fluency; BAC symbol coding; CPT-IP d' total score; HVLT total recall; LNS; NAB Mazes; TMT-A; WMS-III: Spatial Span | 6 |
| van Veelen2010^89^ | Netherlands & Belgium | 74 | 0 | 23.91 (4.42) | 2 | Category fluency; CVLT immediate recall; CVLT 1-5; CVLT long delay free recall;  Letter fluency; Stroop interference; TMT-A; TMT-B; CPT-IP d' total score; WCST perseverative errors; WMS Visual Memory immediate recall; WMS delayed recall | 6 |
| van winkel2006^90^ | Belgium | 80 | 0 | 23.20 (4.00) | 120 | IQ total; IQ verbal; IQ performance; WMS-Paired associated | 6 |
| Zhou2017^91^ | China | 32 | 17 | 25.96 (7.34) | 12 | Category fluency; HVLT total recall; Stroop colour-word | 8 |

COWAT. Controlled Oral Word Association Test; RAVLT. Rey Auditory Verbal Learning Test; ROCF. Rey- Osterrieth Complex Figure Test; TMT. Trail Making Test; WMS. Weschler Memory Scale; LM. logical memory; WCST. Wisconsin Card Sorting Test; CVLT. California Verbal Learning Test; WAIS. Wechsler Intelligence Scale; CPT. Continuous Performance; IQ. intelligent quotient; LNST Letter Number Sequencing Test; NAB. Neuropsychological Assessment Battery; MSCEIT. Mayer-Salovey-Caruso Emotional Intelligence Test; HVLT. Hopkins Verbal Learning Test; BVMT Brief Visuospatial Memory Test; BACS Basic Assessment of Cognition Scale; LNS Letter Number Span

# **eTable 6. Variability between FEP and HC at baseline and follow-up**

| **Baseline** |  |  |  |  |  | **Follow-up** |  |  |  |  |
| --- | --- | --- | --- | --- | --- | --- | --- | --- | --- | --- |
| **task** | **value** | **Std.Error** | **DF** | **t-value** | **p-value** | **value** | **Std.Error** | **DF** | **t-value** | **p-value** |
| **TMT-B** | 0.11 | 0.16 | 6.00 | 0.70 | ns | 0.17 | 0.12 | 6.00 | 1.43 | ns |
| **WCST perseverative errors** | 0.53 | 0.10 | 4.00 | 5.54 | **0.0052** | 0.38 | 0.10 | 2.00 | 3.95 | ns |
| **WCST perseverative responses** | 0.19 | 0.10 | 5.00 | 1.86 | ns | -0.06 | 0.21 | 4.00 | -0.28 | ns |
| **WCST categories** | 0.44 | 0.14 | 1.00 | 3.16 | ns | 0.24 | 0.27 | 1.00 | 0.89 | ns |
| **Executive functioning** | 0.40 | 0.09 | 12.00 | 4.38 | **0.0009** | 0.23 | 0.10 | 10.00 | 2.24 | **0.0493** |
| **TMT-A** | 0.72 | 0.12 | 7.00 | 6.06 | **0.0005** | 0.66 | 0.20 | 4.00 | 3.28 | **0.0304** |
| **Stroop word** | 0.46 | 0.09 | 2.00 | 4.88 | **0.0396** | 0.46 | 0.09 | 1.00 | 5.19 | ns |
| **Stroop colour** | 0.40 | 0.18 | 1.00 | 2.24 | ns |  |  |  |  |  |
| **Stroop colour-word** | 0.10 | 0.07 | 1.00 | 1.52 | ns |  |  |  |  |  |
| **WAIS digit symbol** | -0.07 | 0.03 | 2.00 | -2.19 | ns | 0.35 | 0.06 | 2.00 | 6.22 | 0.0249 |
| **COWAT** | 0.06 | 0.14 | 2.00 | 0.44 | ns | 0.20 | 0.08 | 2.00 | 2.57 | **0.0000** |
| **Processing speed** | 0.51 | 0.14 | 14.00 | 3.53 | **0.0034** | 0.35 | 0.05 | 11.00 | 6.52 | ns |
| **Reasoning and problem solving** | 0.50 | 0.03 | 2.00 | 1.54 | **0.0042** | 0.08 | 0.12 | 1.00 | 0.65 | ns |
| **Social cognition** | 0.14 | 0.14 | 1.00 | 1.03 | ns | 1.70 | 0.42 | 1.00 | 4.16 | ns |
| **CVLT 1-5** | 0.53 | 0.13 | 1.00 | 4.15 | ns | 0.47 | 0.12 | 1.00 | 4.10 | ns |
| **CVLT immediate recall** | 0.57 | 0.16 | 2.00 | 3.60 | ns | 0.86 | 0.18 | 2.00 | 4.69 | **0.0425** |
| **HVLT** | 0.29 | 0.10 | 3.00 | 3.07 | ns | 0.28 | 0.07 | 2.00 | 3.74 | **0.0006** |
| **Logical memory immediate recall** | -0.04 | 0.35 | 2.00 | -0.11 | ns | 0.17 | 0.21 | 2.00 | 0.82 | ns |
| **RVLT** | -0.22 | 0.50 | 1.00 | -0.44 | ns |  |  |  |  |  |
| **Verbal learning** | 0.44 | 0.08 | 12.00 | 5.40 | **0.000160** | 0.49 | 0.09 | 9.00 | 5.46 | **0.0400** |
| **CVLT delayed recall** | 0.07 | 0.26 | 1.00 | 0.26 | ns | 0.12 | 0.21 | 1.00 | 0.58 | ns |
| **Logical memory delayed** | 0.87 | 12.31 | 1.00 | 0.70 | ns | 0.31 | 0.25 | 1.00 | 1.24 | ns |
| **RVLT delayed** | 0.54 | 0.15 | 2.00 | 3.68 | ns |  |  |  |  |  |
| **Verbal memory** | 0.51 | 0.10 | 7.00 | 5.28 | **0.0012** | 0.53 | 0.09 | 5.00 | 5.96 | **0.0019** |
| **BVMT-R** | 0.37 | 0.22 | 2.00 | 1.64 | ns | 0.10 | 0.35 | 1.00 | 0.29 | ns |
| **WMS immediate recall** | 0.59 | 0.14 | 1.00 | 4.31 | ns | 0.32 | 0.25 | 1.00 | 1.29 | ns |
| **Visual learning** | 0.52 | 0.14 | 6.00 | 3.74 | **0.0096** | 0.18 | 0.17 | 4.00 | 1.10 | ns |
| **ROCF delayed** | 0.08 | 0.13 | 2.00 | 0.59 | ns |  |  |  |  | ns |
| **WMS visual memory delayed** | 0.89 | 0.12 | 1.00 | 7.20 | ns | 0.45 | 0.27 | 1.00 | 1.67 | ns |
| **Visual memory** | 0.13 | 0.12 | 5.00 | 1.09 | **0.0000** | 0.23 | 0.22 | 2.00 | 1.06 | ns |
| **Letter number sequencing** | 0.19 | 0.16 | 4.00 | -1.25 | **0.0000** | 0.33 | 0.08 | 4.00 | 3.94 | **0.0002** |
| **WAIS backwards digit** | 0.10 | 0.04 | 2.00 | 2.44 | ns | 0.05 | 0.10 | 2.00 | 0.53 | ns |
| **WAIS forward digit** | 0.48 | 0.28 | 1.00 | 1.73 | ns |  |  |  |  | ns |
| **Working memory** | 0.28 | 0.09 | 7.00 | 2.92 | **0.022** | 0.11 | 0.05 | 5.00 | 2.30 | ns |

ns not significative;

# **eFigure 1. Comparison between FEP and HC at baseline**

**
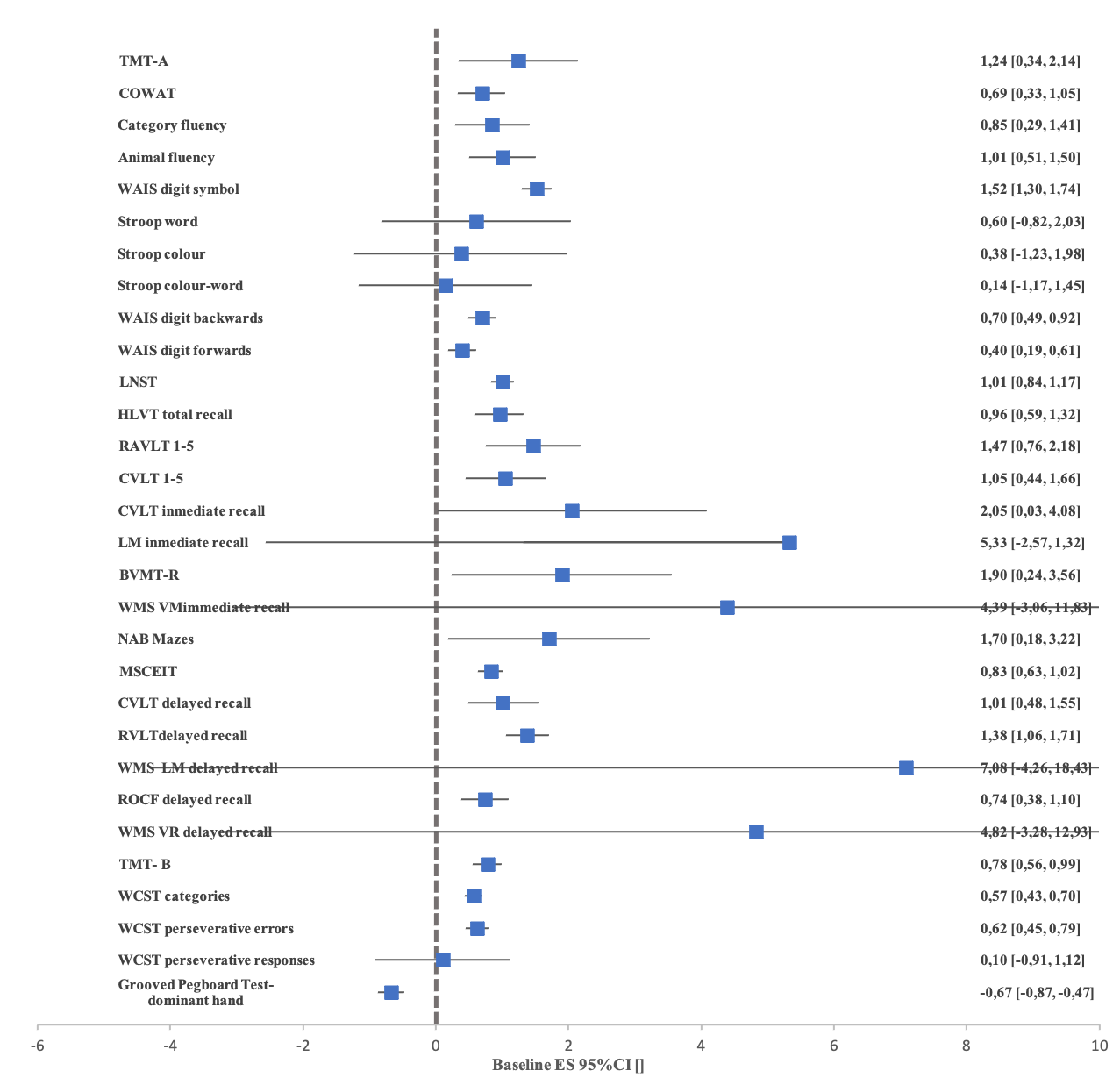
**

# **eTable 7**. Meta-analytical comparisons: FEP vs HC at baseline

FEP First Episode Psychosis; HC. Healthy Controls

| **Meta analysis** | **Tasks** | **k** | **FEP** | **HC** | **ES** | **SE** | **CI95%** | | **z** | **p** | **Heterogeneity** | | | |
| --- | --- | --- | --- | --- | --- | --- | --- | --- | --- | --- | --- | --- | --- | --- |
|  |  |  |  |  |  |  | **lower** | **upper** |  |  | **Q** | **I^2^** | **p** | **trim and fill bias** |
| **Processing speed** | **TMT-A** | 9 | 796 | 491 | 1.24 | 0.46 | 0.34 | 2.14 | 2.70 | **0.0069** | 225.32 | 97.69 | < .0001 | 1.6053 0.3928 4.0862 <.0001 0.8353 2.3752 *** |
|  | **COWAT** | 4 | 379 | 198 | 0.69 | 0.18 | 0.33 | 1.05 | 3.78 | **0.0002** | 9.20 | 68.89 | 0.03 | no |
|  | **Category fluency** | 3 | 167 | 153 | 0.85 | 0.29 | 0.29 | 1.41 | 2.96 | **0.0030** | 9.35 | 78.65 | 0.01 | 1.2991 0.3375 3.8493 0.0001 0.6376 1.9606 *** |
|  | **Animal fluency** | 3 | 217 | 221 | 1.01 | 0.25 | 0.51 | 1.50 | 3.96 | **<.0001** | 6.41 | 75.33 | 0.04 | no |
|  | **WAIS digit symbol** | 4 | 321 | 157 | 1.52 | 0.11 | 1.30 | 1.74 | 13.58 | **<.0001** | 2.16 | 0.00 | 0.54 | 1.3950 0.1053 13.2506 <.0001 1.1887 1.6014 *** |
|  | **Stroop word** | 4 | 245 | 196 | 0.60 | 0.73 | -0.82 | 2.03 | 0.83 | 0.4065 | 153.30 | 97.46 | < .0001 | 0.2268 0.6845 0.3313 0.7404 -1.1149 1.5685 |
|  | **Stroop colour** | 3 | 235 | 163 | 0.38 | 0.82 | -1.23 | 1.98 | 0.46 | 0.6798 | 115.93 | 97.70 | < .0001 | no |
|  | **Stroop colour-word** | 3 | 320 | 268 | 0.14 | 0.67 | -1.17 | 1.45 | 0.21 | 0.8359 | 129.77 | 97.89 | < .0001 | no |
| **Working memory** | **WAIS digit backwards** | 6 | 402 | 357 | 0.70 | 0.11 | 0.49 | 0.92 | 6.41 | **<.0001** | 9.31 | 44.40 | 0.10 | no |
|  | **WAIS digit forwards** | 4 | 238 | 219 | 0.40 | 0.11 | 0.19 | 0.61 | 3.66 | **0.0003** | 3.15 | 14.45 | 0.37 | no |
|  | **LNST** | 6 | 442 | 393 | 1.01 | 0.09 | 0.84 | 1.17 | 11.67 | **<.0001** | 7.18 | 17.66 | 0.21 | no |
| **Verbal learning** | **HLVT total recall** | 5 | 181 | 180 | 0.96 | 0.19 | 0.59 | 1.32 | 5.13 | **<.0001** | 10.14 | 60.47 | 0.04 | 0.8543 0.1920 4.4495 <.0001 0.4780 1.2307 *** |
|  | **RAVLT 1-5** | 3 | 411 | 234 | 1.47 | 0.36 | 0.76 | 2.18 | 4.04 | **<.0001** | 23.68 | 92.65 | < .0001 | no |
|  | **CVLT 1-5** | 3 | 119 | 70 | 1.05 | 0.31 | 0.44 | 1.66 | 3.39 | **0.0007** | 7.15 | 69.27 | 0.03 | no |
|  | **CVLT inmediate recall** | 4 | 307 | 263 | 2.05 | 1.03 | 0.03 | 4.08 | 1.99 | **0.0470** | 99.38 | 98.79 | < .0001 | no |
|  | **LM inmediate recall** | 4 | 404 | 158 | 5.33 | 4.03 | -2.57 | 1.32 | 1.32 | 0.1858 | 53.54 | 99.91 | < .0001 | no |
| **Visual learning** | **BVMT-R** | 5 | 249 | 262 | 1.90 | 0.85 | 0.24 | 3.56 | 2.24 | **0.0253** | 168.68 | 98.17 | < .0001 | no |
|  | **WMS VM immediate recall** | 3 | 116 | 91 | 4.39 | 3.80 | -3.06 | 11.83 | 1.15 | 0.2483 | 51.71 | 99.77 | < .0001 | no |
| **Reasoning and Problem Solving** | **NAB Mazes** | 4 | 190 | 234 | 1.70 | 0.77 | 0.18 | 3.22 | 2.19 | **0.0284** | 117.91 | 97.47 | < .0001 | 2.0633 0.7017 2.9403 0.0033 0.6879 3.4387 ** |
| **Social cognition** | **MSCEIT** | 3 | 221 | 235 | 0.83 | 0.10 | 0.63 | 1.02 | 8.42 | **<.0001** | 0.73 | 0.00 | 0.69 | no |
| **Verbal memory** | **CVLT delayed recall** | 3 | 257 | 213 | 1.01 | 0.27 | 0.48 | 1.55 | 3.75 | **0.0002** | 10.34 | 81.87 | 0.01 | no |
|  | **RVLT delayed recall** | 5 | 618 | 267 | 1.38 | 0.16 | 1.06 | 1.71 | 8.39 | **<.0001** | 14.67 | 72.55 | 0.01 | no |
|  | **WMS LM delayed recall** | 3 | 353 | 192 | 7.08 | 5.79 | -4.26 | 18.43 | 1.22 | 0.2209 | 50.95 | 99.93 | < .0001 | no |
| **Visual memory** | **ROCF delayed recall** | 4 | 547 | 229 | 0.74 | 0.18 | 0.38 | 1.10 | 4.03 | **<.0001** | 14.96 | 76.79 | 0.00 | 0.8293 0.1637 5.0642 <.0001 0.5083 1.1502 *** |
|  | **WMS VR delayed recall** | 3 | 116 | 91 | 4.82 | 4.13 | -3.28 | 12.93 | 1.17 | 0.2436 | 51.01 | 99.80 | < .0001 | no |
| **Executive function** | **TMT- B** | 8 | 611 | 373 | 0.78 | 0.11 | 0.56 | 0.99 | 7.01 | **<.0001** | 14.17 | 48.76 | 0.05 | no |
|  | **WCST categories** | 6 | 626 | 353 | 0.57 | 0.07 | 0.43 | 0.70 | 8.08 | **<.0001** | 3.06 | 0.00 | 0.69 | no |
|  | **WCST perseverative errors** | 7 | 627 | 337 | 0.62 | 0.09 | 0.45 | 0.79 | 7.16 | **<.0001** | 10.12 | 21.78 | 0.12 | no |
|  | **WCST perseverative responses** | 3 | 152 | 186 | 0.10 | 0.52 | -0.91 | 1.12 | 0.20 | 0.8415 | 43.17 | 94.52 | < .0001 | no |
| **Motor functioning** | **Grooved Pegboard Test dominant hand** | 3 | 432 | 139 | -0.67 | 0.10 | -0.87 | -0.47 | -6.63 | **<.0001** | 0.17 | 0.00 | 0.92 | -0.6541 0.0928 -7.0457 <.0001 -0.8361 -0.4722 *** |

# **eTable 8**. Meta-analytical comparisons: FEP vs HC at follow-up.

FEP First Episode Psychosis; HC. Healthy Controls

| **Meta analysis** |  | **k** | **FEP** | **HC** | **ES** | **SE** | **CI95%** | | **z** | **p** | **Heterogeneity** | | | |
| --- | --- | --- | --- | --- | --- | --- | --- | --- | --- | --- | --- | --- | --- | --- |
|  |  |  |  |  |  |  | **lower** | **upper** |  |  | **Q** | **I** | **p** | **trim and fill bias** |
| **Processing speed** | **TMT-A** | 6 | 796 | 491 | 0.79 | 0.19 | 0.41 | 1.17 | 4.09 | <.0001 | 17.58 | 75.69 | **0.0035** | no |
|  | **COWAT** | 4 | 379 | 198 | 0.77 | 0.10 | 0.58 | 0.96 | 7.92 | <.0001 | 1.27 | 0.00 | 0.7349 | 0.7335 0.0901 8.1434 <.0001 0.5569 0.9100 *** |
|  | **Category fluency** | 3 | 167 | 153 | 1.14 | 0.12 | 0.90 | 1.38 | 9.27 | <.0001 | 1.99 | 0.01 | 0.3679 | 1.1445 0.1234 9.2759 <.0001 0.9026 1.3863 *** |
|  | **WAIS digit symbol** | 4 | 321 | 157 | 1.79 | 0.30 | 1.21 | 2.37 | 6.06 | <.0001 | 17.89 | 84.19 | **0.0005** | no |
|  | **Stroop word** | 4 | 245 | 196 | 1.21 | 0.15 | 0.92 | 1.51 | 7.98 | <.0001 | 2.22 | 0.00 | 0.3288 | 1.1554 0.1449 7.9738 <.0001 0.8714 1.4394 *** |
|  | **Stroop colour** | 3 | 342 | 288 | 0.56 | 0.33 | -0.10 | 1.21 | 1.67 | 0.0954 | 10.66 | 82.36 | **0.0049** | no |
| **Working semory** | **WAIS digit backwards** | 5 | 273 | 257 | 0.67 | 0.19 | 0.30 | 1.04 | 3.51 | 0.0004 | 13.10 | 73.14 | **0.0108** | no |
|  | **LNST** | 6 | 442 | 393 | 1.19 | 0.37 | 0.46 | 1.92 | 3.19 | 0.00 | 60.11 | 95.27 | **< .0001** | no |
| **Verbal learning** | **HLVT total recall** | 4 | 131 | 92 | 0.84 | 0.15 | 0.56 | 1.13 | 5.79 | <.0001 | 1.68 | 0.00 | 0.64 | 0.9141 0.1315 6.9516 <.0001 0.6564 1.1718 *** |
|  | **CVLT 1-5** | 3 | 119 | 70 | 1.02 | 0.17 | 0.70 | 1.34 | 6.17 | <.0001 | 1.96 | 4.27 | 0.3747 | 0.7882 0.1890 4.1701 <.0001 0.4178 1.1587 *** |
|  | **CVLT inmediate recall** | 4 | 307 | 263 | 1.71 | 0.74 | 0.27 | 3.16 | 2.32 | 0.02 | 68.46 | 97.80 | **< .0001** | no |
|  | **Logical memory inmediate recall** | 4 | 404 | 158 | 5.32 | 3.90 | -2.32 | 12.97 | 1.36 | 0.17 | 61.09 | 99.90 | **< .0001** | no |
| **Visual learning** | **BVMT-R** | 3 | 209 | 182 | 1.37 | 0.51 | 0.37 | 2.36 | 2.70 | 0.01 | 22.75 | 90.92 | **< .0001** | no |
|  | **WMS VM inmedaite recall** | 3 | 116 | 91 | 1.37 | 0.72 | -0.05 | 2.78 | 1.89 | 0.06 | 15.25 | 99.66 | **0.001** | no |
| **Reasoning and Problem Solving** | **NAB Mazes** | 3 | 114 | 104 | 0.81 | 0.28 | 0.25 | 1.36 | 2.84 | 0.00 | 7.95 | 72.86 | **0.02** | no |
| **Social cognition** | **MSCEIT** | 3 | 207 | 197 | 0.63 | 0.33 | -0.02 | 1.27 | 1.91 | 0.0558 | 14.29 | 83.03 | **0.001** | no |
| **Verbal memory** | **CVLT delayed recall** | 3 | 257 | 213 | 1.02 | 0.17 | 0.70 | 1.35 | 6.15 | <.0001 | 4.25 | 53.08 | 0.1195 | no |
|  | **WMS- LM delayed recall** | 3 | 353 | 192 | 6.38 | 5.17 | -3.75 | 16.52 | 1.23 | 0.22 | 48.08 | 99.91 | **< .0001** | no |
| **Visual memory** | **WMS VR Delayed Recall** | 3 | 116 | 91 | 5.52 | 4.80 | -3.90 | 14.93 | 1.15 | 0.25 | 49.76 | 99.86 | **< .0001** | no |
| **Executive function** | **TMT- B** | 8 | 611 | 373 | 0.69 | 0.12 | 0.45 | 0.94 | 5.64 | <.0001 | 15.73 | 58.78 | **0.03** | no |
|  | **WCST categories** | 4 | 348 | 264 | 0.50 | 0.10 | 0.30 | 0.69 | 5.03 | <.0001 | 4.10 | 19.39 | 0.2505 | no |
|  | **WCST perseverative errors** | 6 | 380 | 271 | 0.55 | 0.19 | 0.18 | 0.91 | 2.93 | 0.00 | 15.71 | 77.61 | **0.0077** | no |
|  | **WCST perseverative responses** | 3 | 152 | 186 | 0.03 | 0.60 | -1.15 | 1.21 | 0.05 | 0.96 | 53.22 | 95.88 | **< .0001** | no |

# **eFigure 2. Comparison between FEP and HC at follow-up**

**
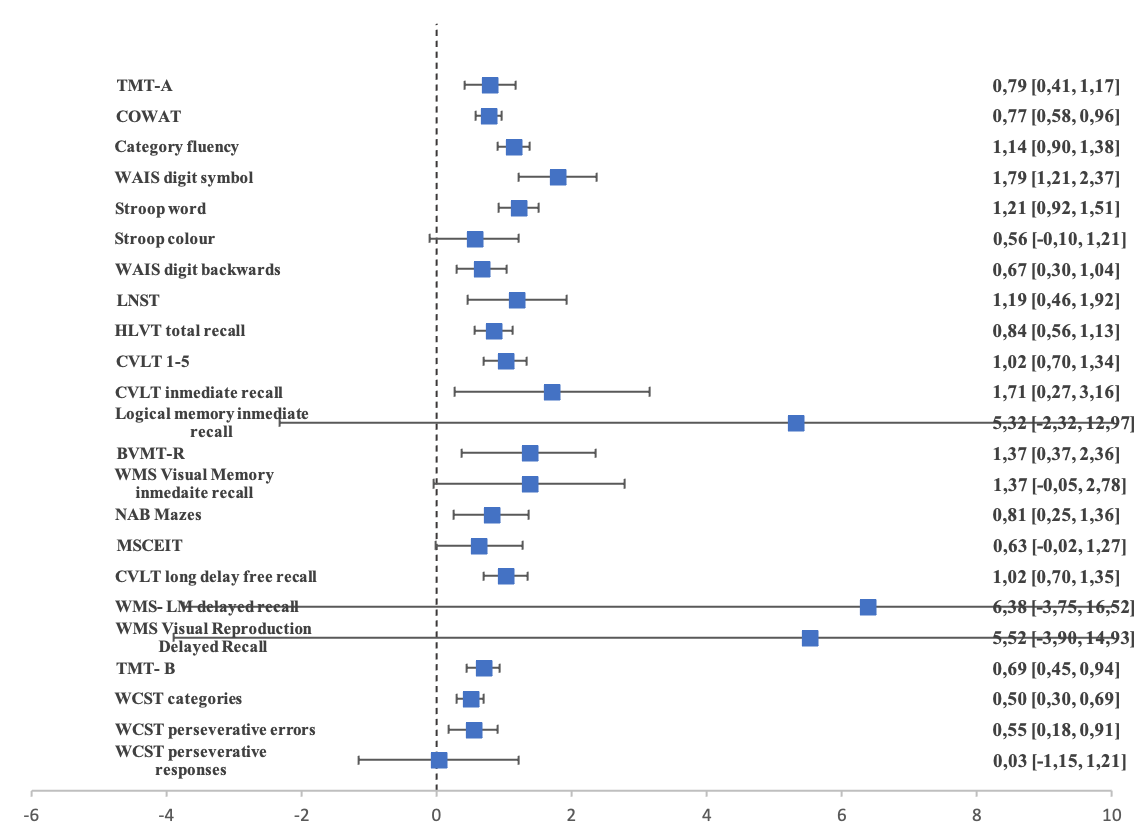
**

# **eTable 9. Comparisons between FEP and HC domains merged**

| **BASALINE** |
| --- |

| **Meta analysis** | **k** | **FEP** | **HC** | **ES** | **SE** | **CI95%** | | **z** | **p** | **Heterogeneity** | | | |
| --- | --- | --- | --- | --- | --- | --- | --- | --- | --- | --- | --- | --- | --- |
|  |  |  |  |  |  | **lower** | **upper** |  |  | **Q** | **I** | **p** | **trim and fill bias** |
| **Processing Speed** | 15 | 1204 | 859 | 0.91 | 0.13 | 0.65 | 1.17 | 6.77 | **<.0001** | 7.12 | 0 | 0.8959 | no |
| **Working Memory** | 9 | 637 | 610 | 0.74 | 0.16 | 0.43 | 1.05 | 4.65 | **<.0001** | 3.36 | 0 | 0.9097 | no |
| **Verbal Learning** | 11 | 638 | 476 | 1.74 | 0.44 | 0.89 | 2.60 | 3.98 | **<.0001** | 89.67 | 88.85 | < .0001 | no |
| **Visual learning** | 8 | 584 | 391 | 2.75 | 0.79 | 1.20 | 4.30 | 3.48 | **0.0005** | 147.74 | 95.26 | < .0001 | no |
| **Verbal memory** | 10 | 946 | 538 | 3.04 | 0.59 | 1.88 | 4.19 | 5.15 | **<.0001** | 130.77 | 93.12 | < .0001 | no |
| **Visual memory** | 7 | 663 | 320 | 2.56 | 0.71 | 1.17 | 3.95 | 3.61 | **0.0003** | 90.74 | 93.39 | < .0001 | no |
| **Executive function** | 14 | 1111 | 663 | 0.60 | 0.19 | 0.24 | 0.97 | 3.25 | **0.0011** | 26.81 | 51.51 | 0.0132 | no |

| **FU** |
| --- |

| **Meta analysis** | **k** | **FEP** | **HC** | **ES** | **SE** | **CI95%** | | **z** | **p** | **Heterogeneity** | | | |
| --- | --- | --- | --- | --- | --- | --- | --- | --- | --- | --- | --- | --- | --- |
|  |  |  |  |  |  | **lower** | **upper** |  |  | **Q** | **I** | **p** | **trim and fill bias** |
| Processing Speed | 14 | 1039 | 709 | 1.01 | 0.13 | 0.74 | 1.27 | 7.45 | **<.0001** | 11.05 | 0 | 0.6067 | no |
| Working Memory | 8 | 508 | 510 | 0.81 | 0.18 | 0.45 | 1.17 | 4.43 | **<.0001** | 7.96 | 12.05 | 0.3362 | no |
| Verbal Learning | 11 | 624 | 438 | 1.81 | 0.47 | 0.90 | 2.72 | 3.89 | **0.0001** | 80.61 | 87.60 | < .0001 | no |
| Visual learning | 6 | 261 | 195 | 1.40 | 0.37 | 0.68 | 2.13 | 3.78 | **0.0002** | 14.60 | 65.77 | 0.0122 | no |
| **Verbal memory** | 8 | 720 | 415 | 3.14 | 0.65 | 1.86 | 4.42 | 4.82 | **<.0001** | 59.30 | 88.20 | < .0001 | no |
| **Visual memory** | 4 | 256 | 131 | 4.58 | 1.60 | 1.44 | 7.73 | 2.86 | **0.0042** | 99.35 | 96.98 | < .0001 | no |
| **Executive function** | 13 | 1095 | 647 | 0.49 | 0.21 | 0.08 | 0.90 | 2.36 | **0.0180** | 26.77 | 55.18 | 0.0083 | 0.2611 0.2022 1.2911 0.1967 -0.1353 0.6575 |

# **eTable 10. Metaregressions FEP vs HC**

| **BASELINE** | **k** | **ES** | **SE** | **CI95%** | | **z** | **p** | **Follow-up** | **k** | **ES** | **SE** | **CI95%** | | **z** | **p** |
| --- | --- | --- | --- | --- | --- | --- | --- | --- | --- | --- | --- | --- | --- | --- | --- |
|  |  |  |  | **lower** | **upper** |  |  |  |  |  |  | **lower** | **upper** |  |  |
| **Processing speed** |  |  |  |  |  |  |  |  |  |  |  |  |  |  |  |
| Age | 14 | 0.03 | 0.03 | -0.021 | 0.08 | 1.08 | 0.28 | Age | 14 | 0.04 | 0.03 | 1.49 | 0.14 | -0.01 | 0.09 |
| Male | 14 | 0.00 | 0.02 | -0.03 | 0.04 | 0.29 | 0.77 | Male | 14 | 0.02 | 0.02 | 1.09 | 0.28 | -0.01 | 0.05 |
| Education | 9 | 0.00 | 0.11 | 0.02 | 0.98 | -0.21 | 0.22 | Education | 9 | -0.01 | 0.11 | -0.09 | 0.93 | -0.22 | 0.20 |
| AP baseline | 10 | 0.00 | 0.01 | -0.42 | 0.68 | -0.01 | 0.01 | AP baseline | 10 | 0.00 | 0.01 | -0.01 | 0.01 | -0.45 | 0.65 |
| Follow-up | 14 | 0.00 | 0.00 | -0.01 | 0.01 | 0.70 | 0.49 | Follow-up | 14 | 0.00 | 0.00 | -0.01 | 0.01 | 0.52 | 0.60 |
| Positive symptoms | 6 | 0.00 | 0.03 | -0.06 | 0.06 | -0.11 | 0.91 | Positive symptoms | 6 | 0.05 | 0.04 | -0.02 | 0.12 | 1.33 | 0.18 |
| Negative symptoms | 7 | 0.02 | 0.02 | -0.02 | 0.07 | 0.93 | 0.35 | Negative symptoms | 7 | 0.03 | 0.03 | -0.02 | 0.09 | 1.26 | 0.21 |
| Functioning | 4 | -0.01 | 0.03 | -0.07 | 0.06 | -0.26 | 0.79 | Functioning | 4 | 0.00 | 0.06 | -0.13 | 0.12 | -0.06 | 0.95 |
| NOS | 14 | -0.08 | 0.13 | -0.34 | 0.18 | -0.59 | 0.56 | NOS | 14 | -0.16 | 0.13 | -0.41 | 0.10 | -1.17 | 0.24 |
| **Working Memory** |  |  |  |  |  |  |  |  |  |  |  |  |  |  |  |
| Age | 9 | 0.00 | 0.04 | -0.08 | 0.07 | -0.12 | 0.91 | Age | 8 | 0.01 | 0.04 | -0.07 | 0.09 | 0.23 | 0.82 |
| Male | 9 | 0.02 | 0.04 | -0.06 | 0.09 | 0.50 | 0.62 | Male | 8 | 0.07 | 0.05 | -0.02 | 0.16 | 1.46 | 0.14 |
| Education | 8 | -0.04 | 0.13 | -0.29 | 0.21 | -0.30 | 0.76 | Education | 8 | -0.01 | 0.14 | -0.30 | 0.27 | -0.08 | 0.93 |
| AP baseline | 6 | 0.00 | 0.02 | -0.05 | 0.05 | -0.01 | 0.99 | AP baseline | 5 | 0.00 | 0.02 | -0.04 | 0.05 | 0.16 | 0.88 |
| Follow-up | 9 | 0.01 | 0.02 | -0.03 | 0.04 | 0.30 | 0.76 | Follow-up | 8 | 0.01 | 0.02 | -0.03 | 0.06 | 0.71 | 0.47 |
| Positive symptoms | 5 | 0.02 | 0.03 | -0.03 | 0.07 | 0.86 | 0.39 | Positive symptoms | 4 | 0.09 | 0.04 | 0.02 | 0.16 | 2.42 | 0.02 |
| Negative symptoms | 5 | 0.02 | 0.06 | -0.09 | 0.13 | 0.30 | 0.76 | Negative symptoms | 4 | 0.32 | 0.13 | 0.06 | 0.59 | 2.42 | 0.02 |
| Functioning | 3 | 0.00 | 0.03 | -0.06 | 0.06 | -0.05 | 0.96 | Functioning | 3 | -0.01 | 0.07 | -0.15 | 0.13 | -0.17 | 0.86 |
| NOS | 9 | -0.03 | 0.16 | -0.35 | 0.29 | -0.21 | 0.83 | NOS | 8 | -0.13 | 0.18 | -0.49 | 0.23 | -0.70 | 0.48 |
| **Verbal Learning** |  |  |  |  |  |  |  |  |  |  |  |  |  |  |  |
| Age | 11 | 0.28 | 0.08 | 0.12 | 0.44 | 3.46 | 0.00 | Age | 11 | 0.29 | 0.08 | 0.14 | 0.45 | 3.69 | 0.00 |
| Male | 11 | 0.02 | 0.09 | -0.15 | 0.18 | 0.21 | 0.84 | Male | 11 | 0.07 | 0.09 | -0.11 | 0.25 | 0.77 | 0.44 |
| Education | 8 | 0.20 | 0.26 | -0.31 | 0.72 | 0.77 | 0.44 | Education | 8 | 0.25 | 0.29 | -0.31 | 0.81 | 0.86 | 0.39 |
| AP baseline | 8 | 0.00 | 0.01 | -0.01 | 0.02 | 0.83 | 0.41 | AP baseline | 8 | 0.00 | 0.01 | -0.01 | 0.01 | 0.31 | 0.76 |
| Follow-up | 11 | 0.08 | 0.01 | 0.05 | 0.11 | 5.62 | <.0001 | Follow-up | 11 | 0.08 | 0.01 | 0.06 | 0.10 | 6.67 | <.0001 |
| Positive symptoms | 5 | 0.05 | 0.03 | -0.01 | 0.11 | 1.56 | 0.12 | Positive symptoms | 5 | 0.02 | 0.04 | -0.06 | 0.09 | 0.39 | 0.69 |
| Negative symptoms | 6 | 0.02 | 0.03 | -0.03 | 0.07 | 0.87 | 0.38 | Negative symptoms | 6 | -0.03 | 0.03 | -0.08 | 0.02 | -1.18 | 0.24 |
| Functioning | na |  |  |  |  |  |  | Functioning | 6 | -0.03 | 0.03 | -0.08 | 0.02 | -1.18 | 0.24 |
| NOS | 11 | -1.96 | 0.38 | -2.70 | -1.22 | -5.21 | <.0001 | NOS | 11 | -1.88 | 0.42 | -2.70 | -1.05 | -4.47 | <.0001 |
| **Visual learning** |  |  |  |  |  |  |  |  |  |  |  |  |  |  |  |
| Age | 8 | 0.24 | 0.14 | -0.04 | 0.51 | 1.67 | 0.10 | Age | 6 | 0.09 | 0.08 | -0.07 | 0.24 | 1.10 | 0.27 |
| Male | 7 | 0.17 | 0.19 | -0.21 | 0.55 | 0.87 | 0.38 | Male | 6 | 0.05 | 0.07 | -0.09 | 0.18 | 0.69 | 0.49 |
| Education | 4 | -0.06 | 0.05 | -0.16 | 0.04 | -1.13 | 0.26 | Education | na |  |  |  |  |  |  |
| AP baseline | 6 | 0.02 | 0.02 | -0.02 | 0.05 | 0.93 | 0.35 | AP baseline | 4 | 0.01 | 0.01 | -0.01 | 0.03 | 0.66 | 0.51 |
| Follow-up | 7 | 0.09 | 0.03 | 0.04 | 0.14 | 3.23 | 0.00 | Follow-up | 6 | 0.01 | 0.01 | -0.01 | 0.04 | 1.24 | 0.21 |
| Positive symptoms | 3 | 0.01 | 0.06 | -0.11 | 0.13 | 0.21 | 0.84 | Positive symptoms | na |  |  |  |  |  |  |
| Negative symptoms | 4 | -0.02 | 0.02 | -0.07 | 0.00 | -1.00 | 0.32 | Negative symptoms | 3 | 0.00 | 0.03 | -0.06 | 0.05 | -0.14 | 0.89 |
| Functioning | na |  |  |  |  |  |  | Functioning | na |  |  |  |  |  |  |
| NOS | 8 | -2.57 | 0.75 | -4.04 | -1.11 | -3.45 | 0.00 | NOS | 6 | -0.46 | 0.32 | -1.09 | 0.17 | -1.43 | 0.15 |
| **Verbal memory** |  |  |  |  |  |  |  |  |  |  |  |  |  |  |  |
| Age | 10 | 0.74 | 0.12 | 0.49 | 0.98 | 5.91 | <.0001 | Age | 8 | 0.66 | 0.13 | 0.40 | 0.92 | 4.97 | <.0001 |
| Male | 10 | 0.12 | 0.08 | -0.03 | 0.27 | 1.57 | 0.12 | Male | 8 | 0.16 | 0.08 | -0.01 | 0.33 | 1.90 | 0.06 |
| Education | 5 | 2.84 | 0.79 | 1.28 | 4.40 | 3.57 | 0.00 | Education | 5 | 2.53 | 0.77 | 1.02 | 4.03 | 3.29 | 0.00 |
| AP baseline | 8 | 0.01 | 0.01 | 0.00 | 0.02 | 1.67 | 0.09 | AP baseline | 7 | 0.01 | 0.01 | -0.01 | 0.02 | 0.91 | 0.36 |
| Follow-up | 10 | 0.17 | 0.02 | 0.12 | 0.21 | 7.36 | <.0001 | Follow-up | 8 | 0.16 | 0.03 | 0.11 | 0.21 | 6.26 | <.0001 |
| Positive symptoms | na |  |  |  |  |  |  | Positive symptoms | na |  |  |  |  |  |  |
| Negative symptoms | 3 | -0.01 | 0.03 | -0.07 | 0.05 | -0.28 | 0.78 | Negative symptoms | na |  |  |  |  |  |  |
| Functioning | na |  |  |  |  |  |  | Functioning | na |  |  |  |  |  |  |
| NOS | 10 | -4.35 | 0.68 | -5.68 | -3.02 | -6.42 | <.0001 | NOS | 8 | -3.93 | 0.75 | -5.40 | -2.47 | -5.26 | <.0001 |
| **Visual memory** |  |  |  |  |  |  |  |  |  |  |  |  |  |  |  |
| Age | 7 | 0.83 | 0.16 | 0.51 | 1.15 | 5.09 | <.0001 | Age | 7 | 1.20 | 0.31 | 0.59 | 1.82 | 3.83 | 0.00 |
| Male | 7 | 0.14 | 0.08 | -0.02 | 0.30 | 1.71 | 0.09 | Male | 4 | 0.39 | 0.31 | -0.23 | 1.00 | 1.23 | 0.22 |
| Education | na |  |  |  |  |  |  | Education | na |  |  |  |  |  |  |
| AP baseline | 4 | 0.00 | 0.01 | -0.01 | 0.01 | -0.45 | 0.65 | AP baseline | na |  |  |  |  |  |  |
| Follow-up | 7 | 0.06 | 0.02 | 0.03 | 0.10 | 3.47 | 0.00 | Follow-up | 7 | 0.08 | 0.04 | -0.01 | 0.16 | 18181.00 | 0.07 |
| Positive symptoms | na |  |  |  |  |  |  | Positive symptoms | na |  |  |  |  |  |  |
| Negative symptoms | na |  |  |  |  |  |  | Negative symptoms | na |  |  |  |  |  |  |
| Functioning | na |  |  |  |  |  |  | Functioning | na |  |  |  |  |  |  |
| NOS | 7 | -3.41 | 0.59 | -4.57 | -2.25 | -5.76 | <.0001 | NOS | 7 | -4.84 | 1.14 | -7.07 | -2.62 | -4.27 | <.0001 |
| **Executive function** |  |  |  |  |  |  |  |  |  |  |  |  |  |  |  |
| Age | 14 | 0.01 | 0.03 | -0.06 | 0.07 | 0.16 | 0.87 | Age | 13 | -0.04 | 0.03 | -0.10 | 0.02 | -1.37 | 0.17 |
| Male | 13 | 0.00 | 0.02 | -0.04 | 0.05 | 0.13 | 0.90 | Male | 13 | 0.02 | 0.03 | -0.04 | 0.07 | 0.64 | 0.52 |
| Education | 9 | -0.15 | 0.16 | -0.47 | 0.17 | -0.91 | 0.36 | Education | 8 | -0.15 | 0.19 | -0.52 | 0.21 | -0.83 | 0.41 |
| AP baseline | 9 | 0.00 | 0.01 | -0.02 | 0.01 | -0.43 | 0.66 | AP baseline | 9 | 0.00 | 0.01 | -0.01 | 0.01 | 0.27 | 0.79 |
| Follow-up | 14 | 0.01 | 0.01 | 0.00 | 0.02 | 1.62 | 0.10 | Follow-up | 13 | -0.01 | 0.00 | -0.02 | 0.00 | -1.67 | 0.10 |
| Positive symptoms | 4 | 0.01 | 0.08 | -0.14 | 0.17 | 0.17 | 0.86 | Positive symptoms | 4 | -0.09 | 0.05 | -0.18 | 0.00 | -2.00 | 0.05 |
| Negative symptoms | 5 | 0.03 | 0.04 | -0.06 | 0.11 | 0.62 | 0.54 | Negative symptoms | 5 | -0.02 | 0.04 | -0.09 | 0.05 | -0.47 | 0.64 |
| Functioning | 4 | 0.07 | 0.05 | -0.01 | 0.16 | 1.64 | 0.10 | Functioning | 4 | 0.08 | 0.06 | -0.04 | 0.20 | 1.31 | 0.19 |
| NOS | 14 | -0.33 | 0.18 | -0.69 | 0.04 | -1.77 | 0.08 | NOS | 13 | -0.31 | 0.15 | 0.02 | 0.60 | 2.07 | 0.04 |

# **eTable 11. Meta-analytical comparisons: FEP baseline vs FEP at follow-up.**

FEP First Episode Psychosis

| **Meta analysis** |  | **k** | **FEP** | **FEP_FU** | **ES** | **SE** | **CI95%** | | **z** | **p** | **Heterogeneity** | | | | |
| --- | --- | --- | --- | --- | --- | --- | --- | --- | --- | --- | --- | --- | --- | --- | --- |
|  |  |  |  |  |  |  | **lower** | **upper** |  |  | **Q** | **I** | **p** | **trim and fill bias** |  |
| **Processing Speed** | **TMT-A** | 21 | 1990 | 1659 | 0.24 | 0.07 | 0.0965 | 0.3794 | 3.3 | 0.0010 | 78.06 | 75.76 | < .0001 | yes | 0.0969 0.0773 1.2535 0.2100 -0.0546 0.2483 |
|  | **BACS Symbol Coding** | 3 | 255 | 194 | 0.1470 | 0.0960 | -0.0411 | 0.3352 | 15.318 | 0.1256 | 1.07 | 0 | 0.5867 | yes | 0.0742 0.0817 0.9076 0.3641 -0.0860 0.2344 |
|  | **COWAT** | 9 | 1089 | 1020 | 0.1672 | 0.0516 | 0.0661 | 0.2684 | 3.24 | 0.0012 | 9.16 | 19.35 | 0.3288 | yes | 0.1001 0.0544 1.8392 0.0659 -0.0066 0.2068 . |
|  | **Category fluency** | 7 | 611 | 579 | 0.07 | 0.07 | -0.06 | 0.20 | 1.00 | 0.3164 | 7.21 | 6.72 | 0.3016 | no |  |
|  | **Animal fluency** | 7 | 850 | 787 | 0.1921 | 0.09 | 0.0111 | 0.3731 | 2.08 | 0.0375 | 14.33 | 62.39 | 0.0262 | no |  |
|  | **Letter fluency** | 6 | 682 | 630 | 0.14 | 0.06 | 0.01 | 0.27 | 2.11 | 0.0343 | 7.86 | 16.29 | 0.1641 | yes | 0.1182 0.0690 1.7114 0.0870 -0.0172 0.2535 . |
|  | **WAIS digit symbol** | 5 | 516 | 433 | 0.0800 | 0.2395 | -0.3894 | 0.5494 | 0.3340 | 0.7384 | 57.98 | 91.70 | < .0001 | no |  |
|  | **Stroop word** | 7 | 403 | 387 | 0.0362 | 0.1199 | -0.1987 | 0.2712 | 0.3023 | 0.7624 | 16.50 | 19.36 | 0.0113 | yes | -0.0791 0.1227 -0.6448 0.5190 -0.3195 0.1613 |
|  | **Stroop colour** | 7 | 415 | 399 | 0.0277 | 0.1015 | -0.1713 | 0.2266 | 0.2724 | 0.7853 | 11.47 | 45.36 | 0.0747 | yes | -0.0402 0.0925 -0.4344 0.6640 -0.2215 0.1411 |
|  | **Stroop colour-word** | 8 | 959 | 776 | 0.0060 | 0.0978 | -0.1858 | 0.1977 | 0.0609 | 0.9515 | 28.32 | 74 | 0.0002 | no |  |
| **Attention/Vigilance** | **CPT-IP d' total score** | 8 | 1143 | 994 | 0.2 | 0.09 | 0.01 | 0.38 | 2.12 | 0.034 | 21.67 | 72.65 | 0.0029 | yes | 0.1537 0.0997 1.5413 0.1233 -0.0418 0.3492 |
|  | **CPT-reaction time** | 5 | 548 | 451 | 0.2662 | 0.0642 | 0.1405 | 0.392 | 4.15 | <.0001 | 2.35 | 0 | 0.671 | yes | 0.2424 0.0605 4.0066 <.0001 0.1238 0.3610 *** |
| **Working Memory** | **WAIS digit backwards** | 8 | 759 | 759 | 0.1505 | 0.0515 | 0.0496 | 0.2513 | 2.92 | 0.0034 | 5.99 | 0 | 0.5409 | no |  |
|  | **WAIS digit forwards** | 8 | 564 | 564 | 0.1402 | 0.0762 | -0.01 | 0.29 | 0.2895 | 0.0656 | 10.33 | 32.39 | 0.1703 | no |  |
|  | **WMS-III: Spatial Span** | 3 | 176 | 176 | 0.3558 | 0.1075 | 0.1451 | 0.5665 | 33.093 | 0.0009 | 0.8913 | 0 | 0.6404 | yes | 0.4188 0.0914 4.5823 <.0001 0.2397 0.5980 *** |
|  | **Letter Number Span (LNS)** | 3 | 192 | 192 | -0.046 | 0.3417 | -0.7157 | 0.6237 | -0.1346 | 0.8929 | 15.20 | 89.39 | 0.0005 | no |  |
|  | **LNST** | 7 | 468 | 468 | 0.0897 | 0.2057 | -0.3135 | 0.4928 | 0.4361 | 0.6628 | 53.83 | 83.30 | < .0001 | yes | -0.0888 0.1970 -0.4508 0.6521 -0.4748 0.2972 |
| **Verbal Learning** | **HLVT total recall** | 7 | 309 | 297 | 0.3014 | 0.0821 | 0.1406 | 0.4622 | 3.67 | 0.0002 | 3.82 | 0 | 0.7014 | no |  |
|  | **RAVLT 1-5 trials** | 5 | 864 | 864 | 0.3100 | 0.1399 | 0.0357 | 0.5842 | 2.21 | 0.0268 | 26.59 | 85.87 | < .0001 | no |  |
|  | **CVLT 1-5** | 7 | 413 | 395 | 0.3089 | 0.0734 | 0.1650 | 0.4529 | 4.21 | <.0001 | 7.49 | 4.25 | 0.2776 | yes | 0.3978 0.0885 4.4935 <.0001 0.2243 0.5713 *** |
|  | **CVLT inmediate recall** | 7 | 698 | 586 | 0.3151 | 0.0566 | 0.2041 | 0.4261 | 5.56 | <.0001 | 3.89 | 0 | 0.6919 | no |  |
|  | **Logical memory inmediate recall** | 6 | 559 | 559 | 0.2638 | 0.0601 | 0.1460 | 0.3817 | 4.39 | <.0001 | 4.12 | 0 | 0.5322 | yes | 0.3137 0.0635 4.9419 <.0001 0.1893 0.4381 *** |
|  | **WMS-Paired associated** | 5 | 356 | 338 | 0.4735 | 0.1261 | 0.2264 | 0.7205 | 3.75 | 0.0002 | 9.38 | 58.43 | 0.0522 | yes | 0.5387 0.1205 4.4691 <.0001 0.3024 0.7750 *** |
| **Visual learning** | **BVMT-R** | 6 | 377 | 337 | 0.6750 | 0.4022 | -0.11 | 1.46 | 1.68 | 0.0933 | 107.93 | 95.84 | < .0001 | no |  |
|  | **WMS Visual Memory inmedaite recall** | 10 | 1073 | 956 | 0.2151 | 0.8364 | -1.42 | 2 | 0.2571 | 0.7971 | 296.70 | 99.65 | < .0001 | no |  |
|  | **Benton correct** | 3 | 76 | 76 | -0.1052 | 0.1624 | -0.4235 | 0.2131 | -0.6476 | 0.5172 | 0.2285 | 0 | 0.8920 | yes | -0.1850 0.1156 -1.6000 0.1096 -0.4115 0.0416 |
|  | **Benton errors** | 3 | 76 | 76 | 0.0152 | 0.1625 | -0.3032 | 0.3337 | 0.0938 | 0.9253 | 0.89 | 0 | 0.6388 | yes | -0.1850 0.1156 -1.6000 0.1096 -0.4115 0.0416 |
| **Reasoning and Problem Solving** | **NAB Mazes** | 6 | 401 | 302 | 0.8001 | 0.4614 | -0.1042 | 17.045 | 1.73 | 0.0829 | 129.12 | 96.44 | < .0001 | no |  |
| **Social cognition** | **MSCEIT** | 6 | 467 | 442 | 0.1337 | 0.0869 | -0.0366 | 0.3040 | 1.54 | 0.1238 | 7.80 | 30.23 | 0.1678 | yes | 0.0621 0.0946 0.6565 0.5115 -0.1233 0.2475 |
| **IQ** | **Wechsler Intelligence Scales full** | 6 | 295 | 260 | 0.2702 | 0.0943 | 0.0854 | 0.4550 | 2.86 | 0.0042 | 5.02 | 15.81 | 0.4125 | yes | 0.1955 0.0898 2.1767 0.0295 0.0195 0.3716 * |
|  | **Wechsler Intelligence Scales verbal** | 5 | 393 | 352 | 0.2445 | 0.1170 | 0.0152 | 0.4737 | 2.09 | 0.0366 | 4.68 | 0.3217 | 0.3217 | yes | 0.2794 0.0674 4.1458 <.0001 0.1473 0.4115 *** |
|  | **Wechsler Intelligence Scales performance** | 4 | 251 | 210 | 0.4416 | 0.0950 | 0.2555 | 0.6278 | 4.65 | <.0001 | 0.5632 | 0 | 0.9048 | yes | 2.1182 2.1985 0.9635 0.3353 -2.1908 6.4272 |
| **Visuospatial ability** | **WAIS Block Design** | 5 | 303 | 303 | 0.1333 | 0.1116 | -0.0855 | 0.3521 | 1.19 | 0.2324 | 5.66 | 37.28 | 0.2260 | yes | 0.1159 0.1025 1.1306 0.2582 -0.0850 0.3169 |
| **Verbal memory** | **CVLT long delay free recall** | 5 | 632 | 520 | 0.2963 | 0.0598 | 0.1792 | 0.4135 | 4.96 | <.0001 | 0.7419 | 0 | 0.9461 | no |  |
|  | **RVLT long delay free recall** | 5 | 877 | 841 | 0.3368 | 0.0571 | 0.22 | 0.4486 | 5.90 | <.0001 | 4.50 | 20.03 | 0.3430 | yes | 0.2932 0.0560 5.2335 <.0001 0.1834 0.4031 *** |
|  | **WMS- LM delayed recall** | 6 | 480 | 480 | 0.1679 | 0.1159 | -0.0593 | 0.3950 | 1.45 | 0.1475 | 10.88 | 56.78 | 0.0537 | yes | 0.3355 0.1371 2.4465 0.0144 0.0667 0.6043 * |
| **Visual memory** | **ROCF Delayed Recall** | 5 | 601 | 601 | 0.1014 | 0.0883 | -0.0716 | 0.2744 | 1.15 | 0.2508 | 7.37 | 49.50 | 0.1177 | no |  |
|  | **WMS Visual Reproduction Delayed Recall** | 7 | 824 | 621 | 0.1971 | 0.1289 | -0.0556 | 0.4498 | 1.53 | 0.1264 | 36.69 | 79.58 | < .0001 | no |  |
| **Executive function** | **TMT- B** | 19 | 1826 | 1578 | 0.4235 | 0.2027 | 0.0262 | 0.8208 | 2.08 | 0.0367 | 545.21 | 96.61 | < .0001 | no |  |
|  | **WCST categories** | 13 | 1616 | 1508 | 0.2335 | 0.036 | 0.1628 | 0.3041 | 6.47 | <.0001 | 10.05 | 0 | 0.6115 | yes | 0.2028 0.0340 5.9568 <.0001 0.1361 0.2695 *** |
|  | **WCST n. errors** | 3 | 206 | 159 | 0.0321 | 0.3877 | -0.7277 | 0.7919 | 0.0827 | 0.9341 | 17.39 | 87.96 | 0.0002 | no |  |
|  | **WCST perseverative errors** | 15 | 1653 | 1525 | 0.3608 | 0.1038 | 0.157 | 0.564 | 3.47 | 0.0005 | 62.28 | 85.72 | < .0001 | yes | 0.2340 0.1073 2.1802 0.0292 0.0236 0.4443 * |
|  | **WCST perseverative responses** | 4 | 453 | 359 | 0.2548 | 0.0776 | 0.1027 | 0.4069 | 3.28 | 0.0010 | 62.37 | 84.66 | < .0001 | yes | 0.3307 0.0617 5.3586 <.0001 0.2097 0.4516 *** |
|  | **Strop interference** | 5 | 297 | 279 | 0.2364 | 0.1852 | -0.1266 | 0.5994 | 1.28 | 0.2019 | 13.06 | 73.96 | 0.0110 | no |  |
| **Motor functioning** | **Tapping test-dominant hand** | 4 | 130 | 130 | 0.1316 | 0.2890 | -0.4348 | 0.6981 | 0.4555 | 0.6488 | 13.42 | 79.76 | 0.0038 | no |  |
|  | **Tapping test-non dominant hand** | 4 | 130 | 130 | 0.0310 | 0.2175 | -0.3952 | 0.457 | 0.1424 | 0.8868 | 8.05 | 64.67 | 0.0450 | no |  |
|  | **Tapping test-two hands** | 3 | 229 | 201 | -0.1918 | 0.3043 | -0.7883 | 0.4046 | -0.6303 | 0.5285 | 17.93 | 88.30 | 0.0001 | no |  |
|  | **Grooved Pegboard Test- dominant hand** | 4 | 452 | 452 | -0.0627 | 0.0665 | -0.1932 | 0.0677 | -0.9427 | 0.3459 | 0.5408 | 0.00 | 0.9098 | yes | -0.0481 0.0622 -0.7722 0.4400 -0.1700 0.0739 |
|  | **Grooved Pegboard Test-non dominant hand** | 3 | 312 | 312 | -0.0340 | 0.0801 | -0.1909 | 0.1230 | -0.424 | 0.6716 | 0.1796 | 0 | 0.9141 | no |  |

# **eFigure 3. Comparisons between FEP at baseline vs follow-up domains merged**

FEP First Episode Psychosis

# **eTable 12. Metaregressions FEP baseline vs follow-up**

|  | **k** | **ES** | **SE** | **CI95%** | | **z** | **p** |
| --- | --- | --- | --- | --- | --- | --- | --- |
|  |  |  |  | **lower** | **upper** |  |  |
| **Processing speed** |  |  |  |  |  |  |  |
| Age | 29 | 0.01 | 0.02 | -0.04 | 0.05 | 0.26 | 0.80 |
| Male | 29 | 0.02 | 0.01 | 0.002 | 0.04 | 2.24 | **0.02** |
| Education | 14 | -0.005 | 0.06 | -0.13 | 0.12 | -0.08 | 0.94 |
| AP baseline | 21 | -0.01 | 0.005 | -0.02 | 0.002 | -1.56 | 0.12 |
| Follow-up | 33 | 0.001 | 0.002 | -0.004 | 0.005 | 0.28 | 0.78 |
| Positive symptoms | 11 | 0.0176 | 0.04 | -0.07 | 0.10 | 0.40 | 0.69 |
| Negative symptoms | 12 | -0.01 | 0.03 | -0.08 | 0.06 | -0.32 | 0.75 |
| Functioning | 6 | -0.01 | 0.04 | -0.10 | 0.07 | -0.31 | 0.75 |
| NOS | 33 | -0.04 | 0.10 | -0.24 | 0.16 | -0.40 | 0.69 |
| **Working memory** |  |  |  |  |  |  |  |
| Age | 13 | -0.07 | 0.05 | -0.16 | 0.02 | -1.45 | 0.15 |
| Male | 13 | 0.01 | 0.02 | -0.03 | 0.05 | 0.55 | 0.58 |
| Education | 9 | -0.09 | 0.14 | -0.38 | 0.19 | -0.66 | 0.51 |
| AP baseline | 11 | 0.003 | 0.01 | -0.01 | 0.02 | 0.38 | 0.70 |
| Follow-up | 17 | 0.01 | 0.01 | -0.03 | 0.02 | -0.73 | 0.46 |
| Positive symptoms | 7 | 0.07 | 0.03 | 0.004 | 0.14 | 2.08 | **0.04** |
| Negative symptoms | 7 | 0.02 | 0.08 | -0.14 | 0.18 | 0.21 | 0.83 |
| Functioning | n.a. |  |  |  |  |  |  |
| NOS | 17 | -0.25 | 0.21 | -0.66 | 0.16 | -1.20 | 0.23 |
| **Attention/vigilance** |  |  |  |  |  |  |  |
| Age | 8 | 0.05 | 0.04 | -0.04 | 0.13 | 1.10 | 0.27 |
| Male | n.a. |  |  |  |  |  |  |
| Education | n.a. |  |  |  |  |  |  |
| AP baseline | n.a. |  |  |  |  |  |  |
| Follow-up | 8 | 0.02 | 0.009 | -0.001 | 0.03 | 1.76 | 0.08 |
| Positive symptoms | n.a. |  |  |  |  |  |  |
| Negative symptoms | n.a. |  |  |  |  |  |  |
| Functioning | n.a. |  |  |  |  |  |  |
| NOS | 8 | -0.03 | 0.13 | -0.28 | 0.22 | -0.24 | 0.81 |
| **Executive functioning** |  |  |  |  |  |  |  |
| Age | 30 | -0.03 | 0.03 | -0.10 | 0.04 | -0.87 | 0.38 |
| Male | 29 | -0.01 | 0.01 | -0.03 | 0.02 | -0.59 | 0.55 |
| Education | 14 | 0.13 | 0.20 | -0.26 | 0.51 | 0.65 | 0.52 |
| AP baseline | 19 | 0.004 | 0.01 | -0.009 | 0.02 | 0.58 | 0.56 |
| Follow-up | 30 | -0.0002 | 0.003 | -0.01 | 0.007 | -0.07 | 0.94 |
| Positive symptoms | 11 | 0.04 | 0.03 | -0.01 | 0.10 | 1.59 | 0.11 |
| Negative symptoms | 10 | -0.05 | 0.08 | -0.20 | 0.10 | -0.70 | 0.48 |
| Functioning | n.a. |  |  |  |  |  |  |
| NOS | 30 | -0.03 | 0.18 | -0.39 | 0.33 | -0.18 | 0.86 |
| **General intelligence** |  |  |  |  |  |  |  |
| Age | 7 | -0.06 | 0.04 | -0.14 | 0.02 | -1.50 | 0.15 |
| Male | 8 | -0.02 | 0.03 | -0.09 | 0.04 | -0.66 | 0.51 |
| Education | n.a. |  |  |  |  |  |  |
| AP baseline | 6 | 0.002 | 0.05 | -0.09 | 0.09 | 0.04 | 0.97 |
| Follow-up | 8 | 0.003 | 0.01 | -0.01 | 0.02 | 0.44 | 0.66 |
| Positive symptoms | n.a. |  |  |  |  |  |  |
| Negative symptoms | n.a. |  |  |  |  |  |  |
| Functioning | n.a. |  |  |  |  |  |  |
| NOS | 8 | 0.33 | 0.22 | -0.11 | 0.78 | 1.48 | 0.14 |
| **Motor functioning** |  |  |  |  |  |  |  |
| Age | 9 | 0.06 | 0.07 | -0.07 | 0.20 | 0.97 | 0.33 |
| Male | 9 | -0.002 | 0.02 | -0.05 | 0.04 | -0.09 | 0.93 |
| Education | n.a. |  |  |  |  |  |  |
| AP baseline | n.a. |  |  |  |  |  |  |
| Follow-up | 9 | -0.0007 | 0.008 | -0.02 | 0.01 | -0.09 | 0.93 |
| Positive symptoms | n.a. |  |  |  |  |  |  |
| Negative symptoms | n.a. |  |  |  |  |  |  |
| Functioning | n.a. |  |  |  |  |  |  |
| NOS | 9 | -0.17 | 0.27 | -0.69 | 0.35 | -0.64 | 0.52 |
| **Verbal learning** |  |  |  |  |  |  |  |
| Age | 21 | -0.02 | 0.02 | -0.07 | 0.03 | -0.78 | 0.43 |
| Male | 21 | -0.03 | 0.01 | -0.05 | -0.002 | -2.12 | **0.034** |
| Education | 11 | -0.004 | 0.08 | -0.17 | 0.16 | -0.05 | 0.96 |
| AP baseline | 19 | -0.002 | 0.005 | -0.01 | 0.008 | -0.36 | 0.72 |
| Follow-up | 27 | -0.001 | 0.005 | -0.01 | 0.008 | -0.30 | 0.79 |
| Positive symptoms | 14 | 0.004 | 0.02 | -0.03 | 0.04 | 0.22 | 0.83 |
| Negative symptoms | 15 | 0.02 | 0.02 | -0.005 | 0.06 | 1.62 | 0.10 |
| Functioning | n.a. |  |  |  |  |  |  |
| NOS | 27 | 0.08 | 0.13 | -0.17 | 0.34 | 0.65 | 0.51 |
| **Verbal memory** |  |  |  |  |  |  |  |
| Age | 12 | 0.04 | 0.03 | -0.10 | 0.02 | -1.32 | 0.19 |
| Male | 13 | -0.02 | 0.01 | -0.04 | -0.002 | -2.19 | **0.03** |
| Education | n.a. |  |  |  |  |  |  |
| AP baseline | 11 | 0.004 | 0.005 | -0.005 | 0.014 | 0.87 | 0.39 |
| Follow-up | 15 | -0.01 | 0.004 | -0.02 | -0.001 | -2.21 | **0.03** |
| Positive symptoms | 7 | -0.007 | 0.01 | -0.02 | 0.01 | -1.04 | 0.30 |
| Negative symptoms | 7 | 0.02 | 0.01 | -0.005 | 0.05 | 1.56 | 0.12 |
| Functioning | n.a. |  |  |  |  |  |  |
| NOS | 15 | 0.28 | 0.10 | 0.08 | 0.49 | 2.7124 | **0.01** |
| **Visual learning** |  |  |  |  |  |  |  |
| Age | 13 | 0.18 | 0.08 | 0.03 | 0.34 | 2.3 | **0.02** |
| Male | 15 | 0.04 | 0.04 | -0.03 | 0.12 | 1.04 | 0.30 |
| Education | 8 | 0.16 | 0.32 | -0.50 | 0.79 | 0.48 | 0.63 |
| AP baseline | 10 | 0.004 | 0.009 | -0.01 | 0.02 | 0.41 | 0.68 |
| Follow-up | 19 | 0.003 | 0.005 | -0.01 | 0.01 | 0.62 | 0.53 |
| Positive symptoms | n.a. |  |  |  |  |  |  |
| Negative symptoms | n.a. |  |  |  |  |  |  |
| Functioning | n.a. |  |  |  |  |  |  |
| NOS | 19 | -0.54 | 0.24 | -1.02 | -0.06 | -2.21 | **0.03** |
| Negative symptoms | n.a. |  |  |  |  |  |  |
| Functioning | n.a. |  |  |  |  |  |  |
| NOS | 14 | -0.12 | 0.09 | -0.29 | 0.045 | -1.42 | 0.16 |

# **eFigure 4. Funnel plots**


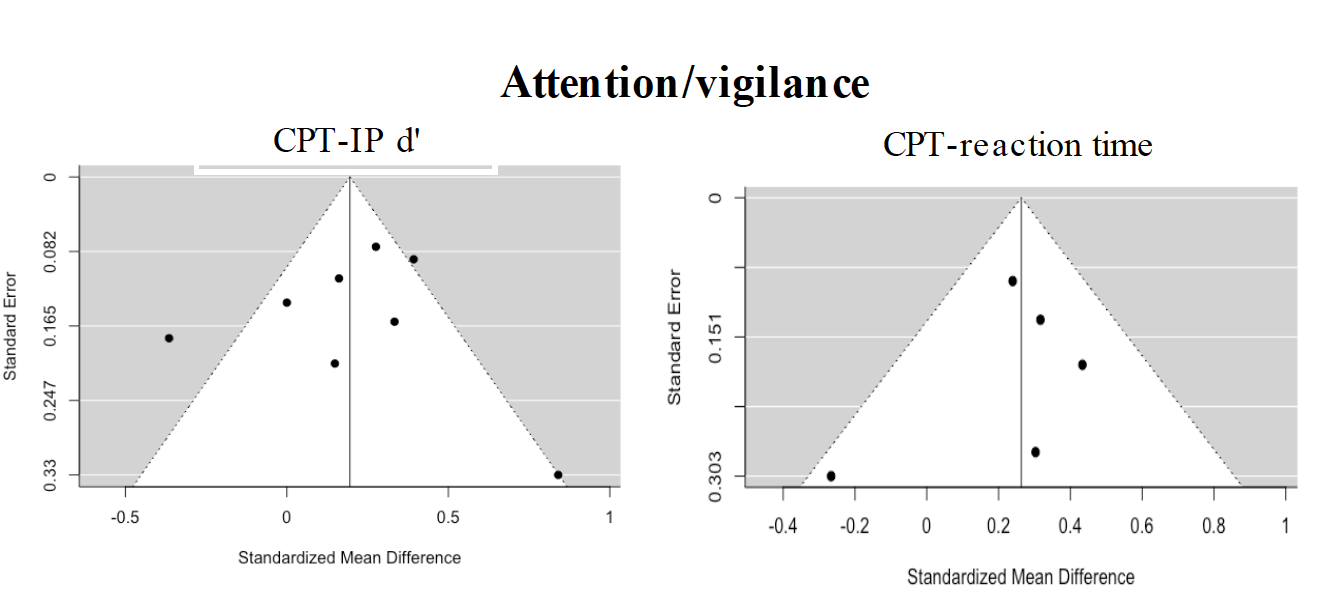

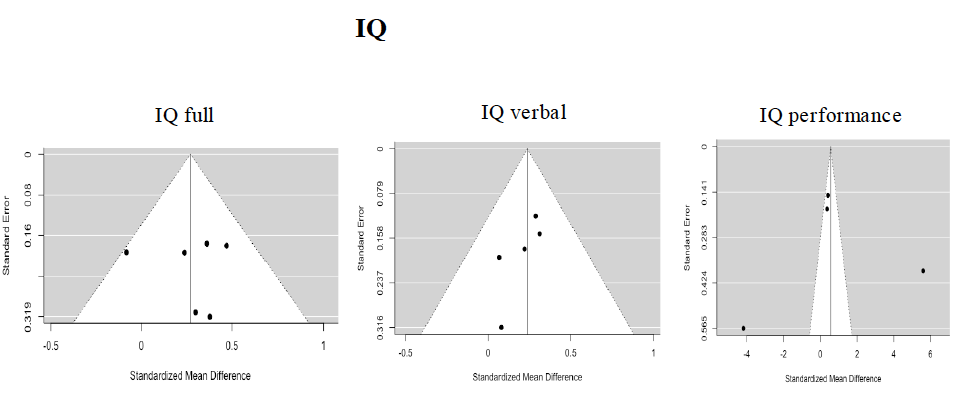

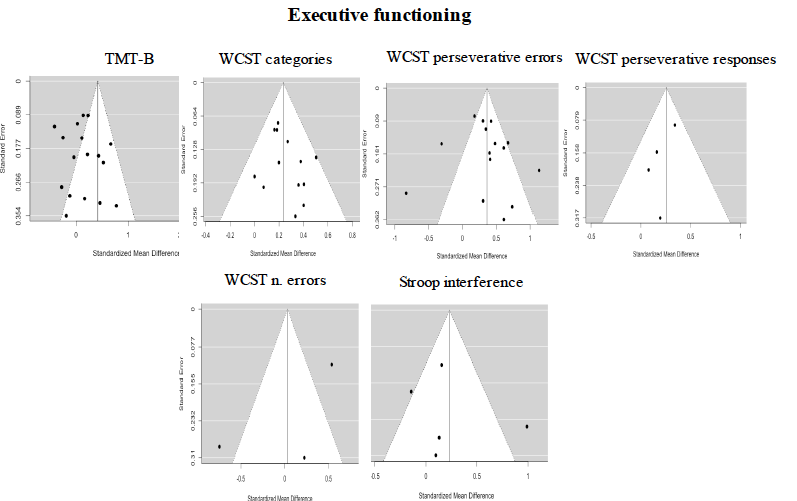


**References**

1. Cochrane Handbook for Systematic Reviews of Interventions. Version 5.1.0. [updated March 2011]. 2011. Available from <www.handbook.cochrane.org>.

2. Borenstein M, Hedges LV, Higgins JPT, Rothstein HR. Part 5. Chapter 22. Complex Data Structures Introduction to Meta-Analysis: John Wiley & Sons, Ltd. ; 2009.

3. Duval S, Tweedie R. Trim and fill: A simple funnel-plot-based method of testing and adjusting for publication bias in meta-analysis. *Biometrics* 2000; **56**(2): 455-63.

4. Seidman LJ, Shapiro DI, Stone WS, et al. Association of Neurocognition With Transition to Psychosis: Baseline Functioning in the Second Phase of the North American Prodrome Longitudinal Study. *JAMA psychiatry* 2016; **73**(12): 1239-48.

5. Westwood SJ, Radua J, Rubia K. Noninvasive brain stimulation in children and adults with attention-deficit/hyperactivity disorder: a systematic review and meta-analysis. *Journal of psychiatry & neuroscience : JPN* 2020; **45**(6): 190179.

6. Norman LJ, Carlisi C, Lukito S, et al. Structural and Functional Brain Abnormalities in Attention-Deficit/Hyperactivity Disorder and Obsessive-Compulsive Disorder: A Comparative Meta-analysis. *JAMA psychiatry* 2016; **73**(8): 815-25.

7. Fusar-Poli P, Deste G, Smieskova R, et al. Cognitive Functioning in Prodromal Psychosis A Meta-analysis. *Archives of General Psychiatry* 2012; **69**(6): 562-71.

8. Hauser M, Zhang J-P, Sheridan EM, et al. Neuropsychological Test Performance to Enhance Identification of Subjects at Clinical High Risk for Psychosis and to Be Most Promising for Predictive Algorithms for Conversion to Psychosis: A Meta-Analysis. *Journal of Clinical Psychiatry* 2017; **78**(1): E28-E40.

9. Reitan RM, Wolfson D. The Halstead–Reitan Neuropsycholgical Test Battery: Therapy and clinical interpretation. Tucson, AZ: Neuropsychological Press.; 1985.

10. Keefe RSE. Brief Assessment of Cognition in Schizophrenia (BACS) Manual—A: Version 2.1. Durham, NC: Duke University Medical Center; 1999.

11. Benton AL, Hamsher K, Sivan AB. Multilingual Aphasia Examination. 3rd ed. ed. Iowa City: IA: AJA Associates; 1994.

12. Benton AL. Differential behavioural effects in frontal lobe disease. . *Neuropsychologia* 1968; **6**: 53–60.

13. Spreen O, Strauss E. A Compendium of Neuropsychological Tests. New York: xford University; 1991.

14. Thurstone LL. Primary mental abilities. *Psychometric Monographs* 1938; **1**.

15. Stroop JR. Studies of interference in serial verbal reactions. . *J Exp Psychol* 1935; **18**: 643-62.

16. Nuechterlei KH, Edell WS, Norris M, Dawson ME. Attentionalvulnerability indicators, thought disorder, and negative symptoms. *Schizophrenia bulletin* 1986; **12**: 408–26.

17. Cornblatt BA, Risch NJ, Faris G, Friedman D, Erlenmeyer-Kimling L. The Continuous Performance Test, Identical Pairs version (CPT-IP), I: new findings about sustained attention in normal families. *Psychiatry research* 1988; **26**: 223-38.

18. Wechsler D. The Wechsler Memory Scale, 3rd ed. . San Antonio, Tex: Psychological Corp (Harcourt); 1997.

19. Gold JM, Carpenter C, Randolph C, Goldberg TE, Weinberger DR. Auditory working memory and Wisconsin Card Sorting Test performance in schizophrenia. *Archives of general psychiatry* 1997; **54**: 159–65.

20. Wechsler D. Wechsler Adult Intelligence Scale—3rd ed (WAISIII): Administration and Scoring Manual. San Antonio, Tex: Psychological Corp; 1997.

21. Wechsler D. Wechsler Adult Intelligence Scale-Fourth Edition (WAIS-IV) APA PsycTests; 2008.

22. Brandt J, R.H.B. B. The Hopkins Verbal Learning Test—Revised: Professional Manual. . Odessa, Fla: Psychological AssessmentcResources, Inc.; 2001.

23. Lezak MD. Neuropsychological Assessment: Oxford University Press; 2004.

24. Woods SP, Delis DC, Scott JC, Kramer JH, Holdnack JA. The California Verbal Learning Test-second edition: Test-retest reliability, practice effects, and reliable change indices for the standard and alternate forms. *Archives of clinical neuropsychology : the official journal of the National Academy of Neuropsychologists* 2006; **21**: 413-20.

25. Delis DC, Kramer JH, Kaplan E, Ober BA. California Verbal Learning Test Manual — II: The Psychological Corporation, San Antonio, TX; 2000.

26. Benedict RHB. Benedict RHB: Brief Visuospatial Memory Test—Revised: Professional Manual. Odessa, Fla: Psychological Assessment Resources, Inc.; 1997.

27. Rey A. L’examen clinique en psychologie. Presses Un, Parisi, M.; 1964.

28. Sivan AB. Benton visual retention test 5th edition ed. San Antonio: Psychological Corporation; 1992.

29. White T, Stern RA. Neuropsychological Assessment Battery: Psychometric and Technical Manual. Lutz, Fla: Psychological Assessment Resources, Inc; 2003.

30. Mayer JD, Salovey P, Caruso DR. Mayer-Salovey Caruso Emotional Intelligence Test: User’s manual. Toronto: ON: Multi-Health Systems.; 2002.

31. Wechsler D. WAIS-R : Wechsler adult intelligence scale-revised. New York, N.Y. : Psychological Corporation; 1981.

32. Wechsler D. Wechsler Intelligence Scale for Children, 3rd edition New York, N. Y,: Psychological Corporation; 1991.

33. Grant DA. Computer version of the Wisconsin Card Sorting Test, WCST. Odessa, Fla: Psychological Assessment Resources; 2000.

34. Stroop JR. Studies of interference in serial verbal reactions. *J Exp Psychol* 1935; **18**: 643-62.

35. Rombouts RP. Finger Tapping Test. In: software] CC, editor.: Lelystad: BuroTesteR; 2002.

36. Roy EA, Square-Storer PA. Neuropsychology of movement sequencing disorders and apraxia. In: Zaidel DW, ed. Neuropsychology. St. Louis: MO: Academic Press; 1994.

37. Addington J, Saeedi H, Addington D. The course of cognitive functioning in first episode psychosis: Changes over time and impact on outcome. *Schizophrenia Research* 2005; **78**(1): 35-43.

38. Albus M, Hubmann W, Scherer J, et al. A prospective 2-year follow-up study of neurocognitive functioning in patients with first-episode schizophrenia. *European Archives of Psychiatry and Clinical Neuroscience* 2002; **252**(6): 262-7.

39. Ayesa-Arriola R, Manuel Rodriguez-Sanchez J, Perez-Iglesias R, et al. Long-term (3-year) neurocognitive effectiveness of antipsychotic medications in first-episode non-affective psychosis: a randomized comparison of haloperidol, olanzapine, and risperidone. *Psychopharmacology* 2013; **227**(4): 615-25.

40. Ayres AdM, Scazufca M, Menezes PR, et al. Cognitive functioning in subjects with recent-onset psychosis from a low-middle-income environment: Multiple-domain deficits and longitudinal evaluation. *Psychiatry Research* 2010; **179**(2): 157-64.

41. Barder HE, Sundet K, Rund BR, et al. Neurocognitive development in first episode psychosis 5 years follow-up: Associations between illness severity and cognitive course. *Schizophrenia Research* 2013; **149**(1-3): 63-9.

42. Barder HE, Sundet K, Rund BR, et al. Ten year neurocognitive trajectories in first-episode psychosis. *Frontiers in Human Neuroscience* 2013; **7**.

43. Burdick KE, Goldberg JF, Harrow M, Faull RN, Malhotra AK. Neurocognition as a stable endophenotype in bipolar disorder and schizophrenia. *Journal of Nervous and Mental Disease* 2006; **194**(4): 255-60.

44. Chan SKW, Chan KKS, Hui CL, et al. Correlates of insight with symptomatology and executive function in patients with first-episode schizophrenia-spectrum disorder: A longitudinal perspective. *Psychiatry Research* 2014; **216**(2): 177-84.

45. Chan RCK, Dai S, Lui SSY, et al. Re-visiting the nature and relationships between neurological signs and neurocognitive functions in first-episode schizophrenia: An invariance model across time. *Scientific Reports* 2015; **5**.

46. Chang WC, Hui CLM, Chan SKW, Lee EHM, Wong GHY, Chen EYH. Relationship between diminished expression and cognitive impairment in first-episode schizophrenia: A prospective three-year follow-up study. *Schizophrenia Research* 2014; **152**(1): 146-51.

47. Crespo-Facorro B, Rodriguez-Sanchez JM, Perez-Iglesias R, et al. Neurocognitive Effectiveness of Haloperidol, Risperidone, and Olanzapine in First-Episode Psychosis: A Randomized, Controlled 1-Year Follow-Up Comparison. *Journal of Clinical Psychiatry* 2009; **70**(5): 717-29.

48. Cuesta MJ, Sanchez-Torres AM, Garcia de Jalon E, et al. Spontaneous Parkinsonism Is Associated With Cognitive Impairment in Antipsychotic-Naive Patients With First-Episode Psychosis: A 6-Month Follow-up Study. *Schizophrenia Bulletin* 2014; **40**(5): 1164-73.

49. Faber G, Smid HGOM, Van Gool AR, Wiersma D, Van den Bosch RJ. The effects of guided discontinuation of antipsychotics on neurocognition in first onset psychosis. *European Psychiatry* 2012; **27**(4): 275-80.

50. Fett A-KJ, Velthorst E, Reichenberg A, et al. Long-term Changes in Cognitive Functioning in Individuals With Psychotic Disorders Findings From the Suffolk County Mental Health Project. *Jama Psychiatry* 2020; **77**(4): 387-96.

51. Frangou S, Hadjulis M, Vourdas A. The Maudsley early onset schizophrenia study: Cognitive function over a 4-year follow-up period. *Schizophrenia Bulletin* 2008; **34**(1): 52-9.

52. Gold S, Arndt S, Nopoulos P, O'Leary DS, Andreasen NC. Longitudinal study of cognitive function in first-episode and recent-onset schizophrenia. *American Journal of Psychiatry* 1999; **156**(9): 1342-8.

53. Gonzalez-Blanch C, Alvarez-Jimenez M, Rodriguez-Sanchez JM, Perez-Iglesias R, Vazquez-Barquero JL, Crespo-Facorro B. Cognitive functioning in the early course of first-episode schizophrenia spectrum disorders - Timing and patterns. *European Archives of Psychiatry and Clinical Neuroscience* 2006; **256**(6): 364-71.

54. Gonzalez-Ortega I, de los Mozos V, Echeburua E, et al. Working memory as a predictor of negative symptoms and functional outcome in first episode psychosis. *Psychiatry Research* 2013; **206**(1): 8-16.

55. Haatveit B, Vaskinn A, Sundet KS, et al. Stability of executive functions in first episode psychosis: One year follow up study. *Psychiatry Research* 2015; **228**(3): 475-81.

56. Hallgren M, Skott M, Ekblom O, Firth J, Schembri A, Forsell Y. Exercise effects on cognitive functioning in young adults with first-episode psychosis: FitForLife. *Psychological Medicine* 2019; **49**(3): 431-9.

57. Harvey PD, Rabinowitz J, Eerdekens M, Davidson M. Treatment of cognitive impairment in early psychosis: A comparison of risperidone and haloperidol in a large long-term trial. *American Journal of Psychiatry* 2005; **162**(10): 1888-95.

58. Hill SK, Schuepbach D, Herbener ES, Keshavan MS, Sweeney JA. Pretreatment and longitudinal studies of neuropsychological deficits in antipsychotic-naive patients with schizophrenia. *Schizophrenia Research* 2004; **68**(1): 49-63.

59. Hoff AL, Svetina C, Shields G, Stewart J, DeLisi LE. Ten year longitudinal study of neuropsychological functioning subsequent to a first episode of schizophrenia. *Schizophrenia Research* 2005; **78**(1): 27-34.

60. Horan WP, Green MF, DeGroot M, et al. Social Cognition in Schizophrenia, Part 2: 12-Month Stability and Prediction of Functional Outcome in First-Episode Patients. *Schizophrenia Bulletin* 2012; **38**(4): 865-72.

61. Jahshan C, Heaton RK, Golshan S, Cadenhead KS. Course of Neurocognitive Deficits in the Prodrorne and First Episode of Schizophrenia. *Neuropsychology* 2010; **24**(1): 109-20.

62. Kenney J, Anderson-Schmidt H, Scanlon C, et al. Cognitive course in first-episode psychosis and clinical correlates: A 4 year longitudinal study using the MATRICS Consensus Cognitive Battery. *Schizophrenia Research* 2015; **169**(1-3): 101-8.

63. Kopala LC, Good KP, Milliken H, et al. Treatment of a first episode of psychotic illness with quetiapine: An analysis of 2 year outcomes. *Schizophrenia Research* 2006; **81**(1): 29-39.

64. Kuharic DB, Makaric P, Kekin I, et al. Changes of neurocognitive status in patients with the first-episode psychosis after 18 months of treatment-A prospective cohort study. *Psychiatry Research* 2021; **304**.

65. Labad J, Barbero JD, Gutierrez-Zotes A, et al. Free thyroxine levels are associated with cognitive changes in individuals with a first episode of psychosis: A prospective 1-year follow-up study. *Schizophrenia Research* 2016; **171**(1-3): 182-6.

66. Leeson VC, Barnes TRE, Hutton SB, Ron MA, Joyce EM. IQ as a predictor of functional outcome in schizophrenia: A longitudinal, four-year study of first-episode psychosis. *Schizophrenia Research* 2009; **107**(1): 55-60.

67. Malla AK, Norman RMG, Manchanda R, Townsend L. Symptoms, cognition, treatment adherence and functional outcome in first-episode psychosis. *Psychological Medicine* 2002; **32**(6): 1109-19.

68. Mayoral M, Zabala A, Robles O, et al. Neuropsychological functioning in adolescents with first episode psychosis: A two-year follow-up study. *European Psychiatry* 2008; **23**(5): 375-83.

69. Minor KS, Friedman-Yakoobian M, Leung YJ, et al. The impact of premorbid adjustment, neurocognition, and depression on social and role functioning in patients in an early psychosis treatment program. *Australian and New Zealand Journal of Psychiatry* 2015; **49**(5): 444-52.

70. Mohn C, Torgalsboen A-K. Details of attention and learning change in first-episode schizophrenia. *Psychiatry Research* 2018; **260**: 324-30.

71. Molina V, Taboada D, Araguees M, Hernandez JA, Sanz-Fuentenebro J. Greater clinical and cognitive improvement with clozapine and risperidone associated with a thinner cortex at baseline in first-episode schizophrenia. *Schizophrenia Research* 2014; **158**(1-3): 223-9.

72. Nopoulos P, Flashman L, Flaum M, Arndt S, Andreasen N. STABILITY OF COGNITIVE-FUNCTIONING EARLY IN THE COURSE OF SCHIZOPHRENIA. *Schizophrenia Research* 1994; **14**(1): 29-37.

73. Olivier MR, Killian S, Chiliza B, et al. Cognitive performance during the first year of treatment in first-episode schizophrenia: a case-control study. *Psychological Medicine* 2015; **45**(13): 2873-83.

74. Pena J, Ojeda N, Segarra R, Ignacio Eguiluz J, Garcia J, Gutierrez M. Executive functioning correctly classified diagnoses in patients with first-episode psychosis: Evidence from a 2-year longitudinal study. *Schizophrenia Research* 2011; **126**(1-3): 77-80.

75. Rodriguez-Sanchez JM, Setien-Suero E, Suarez-Pinilla P, et al. Ten-year course of cognition in first-episode non-affective psychosis patients: PAFIP cohort. *Psychological medicine* 2020: 1-10.

76. Rund BR, Melle I, Friis S, et al. The course of neurocognitive functioning in first-episode psychosis and its relation to premorbid adjustment, duration of untreated psychosis, and relapse. *Schizophrenia Research* 2007; **91**(1-3): 132-40.

77. Saeedi H, Addington J, Addington D. The association of insight with psychotic symptoms, depression, and cognition in early psychosis: A 3-year follow-up. *Schizophrenia Research* 2007; **89**(1-3): 123-8.

78. Sanchez-Torres AM, Moreno-Izco L, Lorente-Omenaca R, et al. Individual trajectories of cognitive performance in first episode psychosis: a 2-year follow-up study. *European Archives of Psychiatry and Clinical Neuroscience* 2018; **268**(7): 699-711.

79. Setien-Suero E, Neergaard K, Ramirez-Bonilla M, et al. Cannabis use in male and female first episode of non-affective psychosis patients: Long-term clinical, neuropsychological and functional differences. *Plos One* 2017; **12**(8).

80. Setien-Suero E, de la Foz VO-G, Suarez-Pinilla P, Crespo-Facorro B, Ayesa-Arriola R. Different neurocognitive profiles of risperidone and aripiprazole in the FIRST episode of psychosis: A 3-year follow-up comparison. *Progress in Neuro-Psychopharmacology & Biological Psychiatry* 2021; **110**.

81. Stirling J, White C, Lewis S, et al. Neurocognitive function and outcome in first-episode schizophrenia: a 10-year follow-up of an epidemiological cohort. *Schizophrenia Research* 2003; **65**(2-3): 75-86.

82. Singh A, Kumar V, Pathak H, et al. Effect of antipsychotic dose reduction on cognitive function in schizophrenia. *Psychiatry Res* 2022; **308**: 114383.

83. The Scottish First Episode Schizophrenia Study V. One-year follow-up. The Scottish Schizophrenia Research Group. *The British journal of psychiatry : the journal of mental science* 1988; **152**: 470-6.

84. Torgalsboen A-K, Mohn C, Rund BR. 2 Neurocognitive predictors of remission of symptoms and social and role functioning in the early course of first-episode schizophrenia. *Psychiatry Research* 2014; **216**(1): 1-5.

85. Torgalsboen A-K, Mohn C, Czajkowski N, Rund BR. Relationship between neurocognition and functional recovery in first-episode schizophrenia: Results from the second year of the Oslo multi-follow-up study. *Psychiatry Research* 2015; **227**(2-3): 185-91.

86. Torrent C, Reinares M, Martinez-Aran A, et al. Affective versus non-affective first episode psychoses: A longitudinal study. *Journal of Affective Disorders* 2018; **238**: 297-304.

87. Townsend LA, Norman RM, Malla AK, Rychlo AD, Ahmed RR. Changes in cognitive functioning following comprehensive treatment for first episode patients with schizophrenia spectrum disorders. *Psychiatry Res* 2002; **113**(1-2): 69-81.

88. Trampush JW, Lencz T, DeRosse P, et al. Relationship of Cognition to Clinical Response in First-Episode Schizophrenia Spectrum Disorders. *Schizophrenia Bulletin* 2015; **41**(6): 1237-47.

89. van Veelen NMJ, Grootens KP, Peuskens J, et al. Short term neurocognitive effects of treatment with ziprasidone and olanzapine in recent onset schizophrenia. *Schizophrenia Research* 2010; **120**(1-3): 191-8.

90. van Winkel R, Myin-Germeys I, Delespaul P, Peuskens J, De Hert M, van Os J. Premorbid IQ as a predictor for the course of IQ in first onset patients with schizophrenia: A 10-year follow-up study. *Schizophrenia Research* 2006; **88**(1-3): 47-54.

91. Zhou F-C, Wang C-Y, Ungvari GS, et al. Longitudinal changes in prospective memory and their clinical correlates at 1-year follow-up in first-episode schizophrenia. *Plos One* 2017; **12**(2).
